# The genomic basis of independent marine transitions in turtles: convergent episodic adaptation and demographic shifts

**DOI:** 10.1093/molbev/msag114

**Published:** 2026-05-06

**Authors:** Elisa Ramos, Leon Hilgers, Tomás Carrasco-Valenzuela, Diego De Panis, Agnello Picorelli, James Sullivan, Marcela Uliano-Silva, Blair P Bentley, Peter A Scott, Michael Hiller, Mariana F Nery, Howard Bradley Shaffer, Lisa Komoroske, Camila J Mazzoni

**Affiliations:** Department of Evolutionary Genetics, Leibniz-Institut für Zoo- und Wildtierforschung (IZW), Berlin, Germany; Department of Genetics, Evolution, Immunology and Microbiology, State University of Campinas, Campinas, SP, Brazil; Department of Comparative Genomics, Senckenberg Research Institute, Senckenberganlage 25, Frankfurt 60325, Germany; Institute of Cell Biology and Neuroscience, Faculty of Biosciences, Goethe University Frankfurt, Max-von-Laue-Str. 9, Frankfurt 60438, Germany; Department of Evolutionary Genetics, Leibniz-Institut für Zoo- und Wildtierforschung (IZW), Berlin, Germany; Institute of Biochemistry and Biology, Universität Potsdam, Brandenburg, Potsdam, Germany; Department of Evolutionary Genetics, Leibniz-Institut für Zoo- und Wildtierforschung (IZW), Berlin, Germany; Department of Genetics, Evolution, Immunology and Microbiology, State University of Campinas, Campinas, SP, Brazil; Department of Evolutionary Genetics, Leibniz-Institut für Zoo- und Wildtierforschung (IZW), Berlin, Germany; Tree of Life, Wellcome Sanger Institute, Cambridge CB10 1SA, UK; Faculty of Biosciences and Aquaculture, Nord University, Bodø, Norway; Department of Biological Sciences, Smith College, Northampton, MA 01063, USA; Natural Sciences Collegium, Eckerd College, 4200 54 Ave S, St Petersburg, FL 33711, USA; Department of Comparative Genomics, Senckenberg Research Institute, Senckenberganlage 25, Frankfurt 60325, Germany; Department of Genetics, Evolution, Immunology and Microbiology, State University of Campinas, Campinas, SP, Brazil; Department of Ecology and Evolutionary Biology, University of California, Los Angeles, CA 90095, USA; La Kretz Center for California Conservation Science, Institute of the Environment and Sustainability, University of California, Los Angeles, CA 90095, USA; Department of Environmental Conservation, University of Massachusetts Amherst, Amherst, MA 01003, USA; Department of Evolutionary Genetics, Leibniz-Institut für Zoo- und Wildtierforschung (IZW), Berlin, Germany

**Keywords:** molecular evolution, natural selection, adaptation, marine turtle

## Abstract

The transition from terrestrial to marine environments represents one of the most fundamental evolutionary shifts in vertebrate history, requiring radical physiological and genomic remodeling. We investigated the genomic signatures of saltwater adaptation in the green sea turtle (*Chelonia mydas*), the leatherback turtle (*Dermochelys coriacea*), and the independently evolved estuarine diamondback terrapin (*Malaclemys terrapin*). Our analyses reveal that the marine transition is characterized by rapid evolution and expansion in gene families linked to iron metabolism, organ morphogenesis, and sensory perception—patterns that mirror those seen in other secondarily marine tetrapods. Notably, while we identified shared targets of positive selection across these independent lineages, we found no evidence of repeated evolution at the nucleotide level, reinforcing that functional convergence often arises through distinct molecular trajectories. Furthermore, demographic reconstructions reveal that saltwater-adapted turtles share a history of deep-time population declines; however, the delayed recovery of *M. terrapin* underscores the specific susceptibility of estuarine specialists to Pleistocene sea-level volatility. By bridging comparative genomics and historical demography, this study provides new insights into the genomic basis of marine adaptations in turtles and a comprehensive framework for understanding the molecular and ecological mechanisms that facilitate major vertebrate transitions into the marine realm.

## Introduction

Adaptation to new environments is a fundamental component of evolutionary diversification (Reznick and Ghalambor 2001; Colautti and Lau 2015; Stroud and Losos 2016). Species entering new environments are often exposed to novel, strong selection pressures, leading to rapid adaptation and the emergence of distinct phenotypes within their expanded range (Reznick and Ghalambor 2001; Stroud and Losos 2016). A classic example of this process is the transition from terrestrial or freshwater ancestors into marine habitats, which requires profound morphological, physiological, and behavioral transformations (Pyenson et al. 2014). This particular environmental shift introduces challenges including, high salinity, oxygen availability, buoyancy regulation, temperature variation, pathogen exposure, and altered sensory environments, all demanding extensive and often coordinated evolutionary responses (Houssaye and Fish 2016; Kelley et al. 2016).

All major amniote lineages have successfully recolonized marine environments, adapting and diversifying in these habitats (Pacini and Harper 2008; Pyenson et al. 2014; Vermeij and Motani 2018; Motani and Vermeij 2021). Genomic analyses of cetaceans have revealed how natural selection reshaped core physiological systems to meet marine environmental demands following the land-water transition, including increased myoglobin stability and abundance to enhance oxygen storage for prolonged dives (Dasmeh et al. 2013), expansions of anaerobic metabolism gene families and cardiovascular adaptations to tolerate extreme hypoxia and high pressures associated with deep diving (McGowen et al. 2012; Sun et al. 2013; Yim et al. 2014), and loss of hair-type keratin genes alongside changes in lipid metabolism enabling blubber-based thermal insulation and streamlining (Nery et al. 2014; Sun et al. 2017; Yuan et al. 2021). Adaptive responses in cetacean sensory genes further reflect the need to navigate and forage effectively underwater given the changes in light and acoustic properties of water compared to land (Keane et al. 2015; Zhou et al. 2018). Comparative genomic studies in penguins, which are secondarily aquatic, flightless diving birds, reveal strikingly similar patterns of molecular adaptation. Penguins show positive selection and accelerated evolution in genes involved with oxygen transport and anaerobic metabolism for deep diving, modifications of forelimb development into rigid flippers, specialized fat deposition for thermoregulation, and visual system tuning for dim marine light environments (Vianna et al. 2020; Cole et al. 2022).

In the saltwater crocodile (*Crocodylus porosus*), adaptations to saline habitats are primarily supported by highly conserved lingual salt glands rich in ion pumps (Cramp et al. 2008). Genomic signatures in this species indicate an enrichment of pathways related to potassium channel pore domains and peroxisomal membranes, reflecting specialized osmoregulatory needs (Ghosh et al. 2020). Notably, key subunits of the Na^+^/K^+^-ATPase cation transporter exhibit strong signals of positive selection, which is associated with the species’ ability to maintain ionic homeostasis via efficient salt excretion in hyperosmotic environments (Ghosh et al. 2020). Similarly, secondarily marine squamates, such as the species-rich Hydrophis sea snake lineage, show genomic signatures reflecting their transition to marine life. These include adaptations for hypoxia tolerance, alpha-keratin modifications that support cutaneous gas exchange (Ludington et al. 2023), and blue-shifted visual pigments adapted to deep-water light environments (Seiko et al. 2020; Simões et al. 2020). These squamates also exhibit adaptations in renal and salt-gland functions that facilitate water balance within hyperosmotic seawater (Peng et al. 2020; Ludington et al. 2023).

Together, these findings demonstrate how consistent selective pressures during the transition to a marine environment drive the same or similar evolutionary solutions in key physiological systems among even distantly related lineages. These comparative genomic studies also highlight both shared and lineage-specific molecular changes, encouraging further investigation of the ways that other secondarily marine lineages, eg the marine turtles, have adapted to the unique challenges of life in the ocean.

Chelonians (order Testudines) represent a lineage of significant evolutionary interest, characterized by a deep divergence from other reptiles tracing back to the Middle Triassic Period (Joyce et al. 2013; Crawford et al. 2015; Irisarri et al. 2017). The fossil record documents the transition toward their unique body plan, beginning with stem-turtles such as *Eunotosaurus* and the partially shelled *Odontochelys semitestacea*, leading to the appearance of the fully shelled crown group in the Late Triassic, approximately 210 to 230 million years ago (Li et al. 2008; Joyce et al. 2013). This ancient origin and highly specialized anatomy made their phylogenetic placement difficult to resolve for decades, with molecular data eventually confirming their position as the sister group to Archosauria (Zardoya and Meyer 2001; Chiari et al. 2012; Shaffer et al. 2013). Modern Testudines included species commonly referred to as turtles, tortoises, and terrapins. These terms are widely used in ecological or regional contexts, but do not always align with formal taxonomy. Tortoises, for example, represent a taxonomically distinct group within Testudines, belonging to the family Testudinidae and adapted to terrestrial environments. Terrapins, in contrast, do not form a monophyletic group and are generally used to describe semi-aquatic species that inhabit freshwater or brackish environments. Turtles, in the broader sense, include all other aquatic species, including those that are fully marine (Lovich and Gibbons 2021; TTWG 2025). While internal relationships within Testudines historically lacked consensus, particularly regarding the placement of marine lineages, molecular phylogenies based on extensive datasets have now provided strong support for the evolutionary relationships within the group (Guillon et al. 2012; Crawford et al. 2015; Shaffer et al. 2017; Thomson et al. 2021).

Marine turtles (Superfamily Chelonioidea) represent a natural experiment for studying secondary adaptation to oceanic life. Although turtles have transitioned between aquatic and terrestrial environments multiple times in their evolution (Evers and Benson 2019), only one extant clade successfully occupies truly oceanic habitats. This group includes the families Dermochelyidae and Cheloniidae and is represented by seven extant species: the leatherback turtle (*Dermochelys coriacea*, the only living member of Dermochelyidae), and six species within Cheloniidae: the green (*Chelonia mydas*), loggerhead (*Caretta caretta*), hawksbill (*Eretmochelys imbricata*), Kemp's ridley (*Lepidochelys kempii*), olive ridley (*Lepidochelys olivacea*), and flatback turtles (*Natator depressus*). In addition, a few coastal lineages, best exemplified by the diamondback terrapin (*Malaclemys terrapin*), are also adapted to saltwater environments (Southwood Williard et al. 2019). Turtles therefore provide a valuable model for exploring how physiological and genomic traits evolve in response to saltwater and marine conditions.

Recent advances in genome sequencing and broader taxon sampling now enable detailed investigations into these transitions, addressing long-standing challenges associated with the deep evolutionary history and distinctive biology of turtles. In particular, genomic studies have already provided important insights into the molecular basis of these evolutionary adaptations. For example, the genomes of the Chinese soft-shelled turtle (*Pelodiscus sinensis*) and the green sea turtle (*C. mydas*), as well as the red-eared slider (*Trachemys scripta*) transcriptome, have revealed the genetic mechanisms behind shell formation and the internalization of the ribcage and limb girdles, an evolutionary innovation unique to turtles among living and extinct tetrapods (Kaplinsky et al. 2013; Wang et al. 2013). The painted turtle (*Chrysemys picta bellii*) genome has provided insights into turtles’ relatively slow rate of molecular evolution compared to other amniotes, as well as traits related to longevity and physiological adaptations to extreme conditions, such as anoxia and freeze tolerance in overwintering hatchlings (Shaffer et al. 2013). Genomic studies of the pinta Island tortoise (*Chelonoidis abingdonii)* and Aldabra tortoises (*Aldabrachelys gigantea*) have identified candidate genes that may influence their remarkable size and longevity (Quesada et al. 2019). Research on amino acid changes linked to the desert tortoise's (*Gopherus agassizii*) adaptations to arid environments have identified genes related to kidney function and skin keratinization that show signs of selection for water conservation (Tollis et al. 2017). Chelonians have relatively long lifespans, and recent studies recovered cellular mechanisms related to resistance to oxidative stress associated with aging in this group (Glaberman et al. 2021). Other efforts have described adaptive patterns in biological systems related to cellular respiration (Duchene et al. 2012; Escalona et al. 2017; Ramos et al. 2020) and anatomical development, particularly genes involved in shell evolution, which is a defining morphological trait in turtles (Wang et al. 2013).

While previous genomic studies have greatly enhanced our understanding of turtle evolution, the genetic basis of adaptations to marine environments in marine turtles remains less explored. Initial studies of marine turtle genomes focused mainly on mitochondrial markers (Duchene et al. 2012; Escalona et al. 2017) or a single marine turtle species (Wang et al. 2013) while population genetic studies have contributed substantially to our understanding of marine turtle migration patterns and associated conservation needs (Rodríguez-Zárate et al. 2013; Chow et al. 2019; Ng et al. 2024). Recently, Bentley et al. (2023) produced high-quality, chromosome-level genome assemblies for two marine turtle species, *C. mydas* and *D. coriacea*. Their analyses revealed unique genetic adaptations in immune and sensory genes, traits that are closely linked to the distinct life histories of marine turtles.

Here, we explore the genomic structure and function of marine turtles in greater depth by analyzing coding regions and identifying evolutionary strategies that contribute to their success in marine habitats. Using a comparative framework spanning terrestrial, freshwater, and saltwater relatives, we test for lineage-specific expansions in gene families, for evidence of positive selection along key marine branches, and for shared molecular adaptations in independently saltwater adapted turtles. We identified genes that may have contributed to the early radiation of the marine turtle clade, testing for signatures of positive selection in the stem branch of Chelonioidea. To provide evolutionary context, we reconstructed the demographic histories of *M. terrapin* and 11 other chelonian species, comparing our results with previously reported demographic profiles for the superfamily Chelonioidea (Bentley et al. 2023; Arantes et al. 2025). These reconstructions allow us to contextualize the timing of lineage diversification and the genomic consequences of marine colonization, with a specific focus on the recent saltwater transition of the estuarine specialist, *M. terrapin*. Together, these analyses provide an integrated view of the genomic strategies underlying marine adaptation in turtles and reveal both shared and lineage-specific evolutionary responses to marine environments (Figs. 3 and 4).

## Results

### Genome data evaluation

We analyzed a high-quality dataset of 17 turtle genomes (selected from an initial 26 publicly available genome assemblies) representing all major superfamilies and diverse ecological niches, including 11 chromosome-level assemblies (see Materials and Methods for further details regarding data sources and assembly versions). To evaluate genome quality across sampled turtle species, we used the Genome Evaluation Pipeline (GEP) (Sullivan et al. 2025) to assess assembly statistics including contiguity, completeness, and base-level accuracy. Assembly levels ranged from contig to chromosome, with total sequence lengths spanning from 1.79 Gb in the hickatee (*Dermatemys mawii*) to 2.48 Gb in *C. picta bellii*. Chromosome-level assemblies generally exhibited superior scaffold N50 values, with *T. scripta elegans*, the chinese pond turtle (*Mauremys reevesii*), and *G. evgoodei* surpassing 139 Mb. Among scaffold-level assemblies, the Red-bellied short-necked turtle (*Emydura subglobosa*) and the pig-nosed turtle (*Carettochelys insculpta*) showed relatively high scaffold N50s (>46 Mb), despite lower contig N50s. Quality values (QV) (Rhie et al. 2020) varied widely, from 26.0 in *C. abingdonii* to 54.9 in *C. insculpta*, reflecting differences in base-level accuracy. The assemblies of both marine turtle species (*C. mydas* and *D. coriacea*) are among the most complete in our dataset. These metrics indicate substantial variability in assembly quality across species, with a subset of genomes suitable for downstream comparative analyses. Assembly quality metrics retrieved by GEP results are summarized in Table S1.

### Gene annotation and lost genes

When available, annotations provided by National Center for Biotechnology Information pipeline (NCBI) typically predicted a higher number of proteins than annotations done with TOGA (Tool to infer Orthologs from Genome Alignments (Kirilenko et al. 2023)). However, TOGA consistently identified around 18,300 proteins across all assemblies, including those without NCBI annotations, allowing for standardized comparisons across species (Table S2). For the genomes with an available annotation on NCBI, TOGA annotated, on average, 3,187 fewer protein-coding genes than the NCBI annotation after removing the lost transcripts (Table S2), suggesting that lost genes could be present on NCBI annotations.

Benchmarking Universal Single-Copy Orthologues (BUSCOs) scores for assemblies and NCBI annotations indicated high completeness across all annotated genomes, with most species recovering over 97% of expected single-copy orthologs (Table S1). BUSCO scores for annotations retrieved from TOGA revealed most genomes recovering over 93% of the expected ortholog and are shown in Figure S1. As expected, TOGA-based annotations show slightly lower completeness scores than species-specific NCBI annotations (Figure S2). However, completeness remained high across all assemblies (mostly above 92%), supporting TOGA's suitability for non-model species. This is particularly true for clades like turtles, which are characterized by slow mutation rates (Shaffer et al. 2013; Brownstein et al. 2024) and highly alignable genomes—both favorable features for TOGA's performance (Kirilenko et al. 2023).

Genes identified as lost by TOGA projections contain gene-inactivating mutations such as premature stop codons or frameshifts in the sequence for the query species. For the two marine species, 1,021 genes were identified as lost in *D. coriacea* and 526 in *C. mydas* in relation to the freshwater generalist *T. scripta elegans*. Table S3 shows the genes classified as lost for all 16 turtle species tested, showing gene losses that are shared among species and the ones that are unique for each turtle species. Twenty-seven genes from the entire dataset were commonly lost in both marine turtle species, but retained in all other taxa. A total of 49 genes were lost exclusively by *C. mydas*, while 328 were lost exclusively by *D. coriacea*. Table S4 shows the exclusive and shared genes classified as lost by *D. coriacea* and *C. mydas* and their respective annotated functions based on *T. scripta elegans* genome annotation. Genes associated with immune response, olfactory and gustatory perception, and antioxidant defense were among those specifically lost in marine turtles (Table S4), although, only the olfactory transduction Gene Ontology (GO) term category showed enrichment of representing genes classified as lost exclusively for both species (Table S5). The lost genes identified in *D. coriacea* and *C. mydas* were spread across the genomes with most chromosomes having at least one lost gene (Table S6).

### Orthology inference

Following assembly evaluation, our selected genome dataset included assemblies from 13 of the 14 recognized Testudine families; no genome assembly was available for Kinosternidae at the time of our analysis. Genome assemblies for eight outgroup species from other vertebrate lineages were also included in our genomic dataset to increase tree resolution, improve support for clades, and help reconstruct the true evolutionary history of each gene and species tree (see Materials and Methods and Table S7). The selected turtle species spanned diverse ecological niches, including two open-ocean marine turtles, eleven freshwater turtles, one inshore estuarine turtle, and three terrestrial turtle species.

Two complementary datasets of protein-coding gene sequences were generated for orthology inference: the *Turtle dataset*, containing 17 turtle species annotated using TOGA projections, and the *Vertebrate dataset*, comprising eight turtle species and all outgroup taxa, all with NCBI annotations (Table S7). From the *Turtle dataset*, we identified 14,897 single-copy orthologs while the *Vertebrate dataset* retrieved 19,701 orthogroups, including 6,000 single-copy orthologs.

### Phylogenetic inference and gene family dynamics

**Figure 1:**
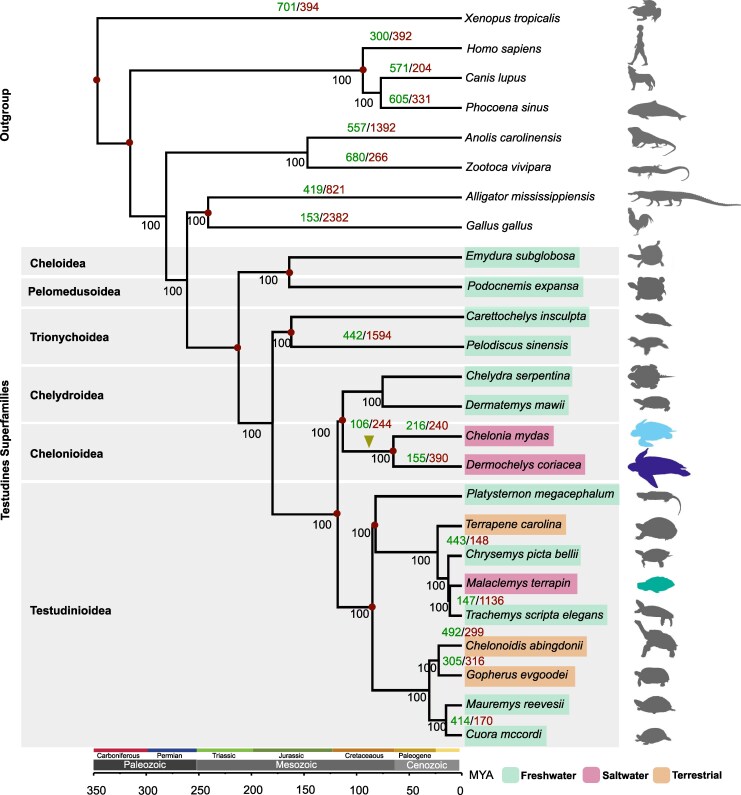
Phylogenetic and gene family dynamics analysis of Testudines. A maximum likelihood (ML) phylogenetic tree was inferred from concatenated protein-coding sequences of 6,000 shared single-copy genes. Species divergence times were estimated using r8s (Sanderson 2003) and are shown in millions of years. Bootstrap values, based on 1,000 ML pseudoreplicates, are indicated below the branches. The tree was calibrated using fossil-based divergence times from Joyce et al. (2013) and Wang et al. (2013), indicated by red dots at the corresponding nodes. Turtle latin binomials are color-coded by habitat: freshwater (mint green), saltwater (pink), and terrestrial (orange). Colored turtle silhouettes denote the three saltwater species: *Chelonia mydas* (light blue), *Dermochelys coriacea* (purple), and *Malaclemys terrapin* (teal); all non-marine species are gray. Numbers above the branches indicate the number of orthogroups (OGs) showing expansion (green) or contraction (red), from a total of 14,452 OGs identified in the *Vertebrate dataset*. The inverted olive triangle marks the SMT. Turtle silhouettes are from the public domain (PhyloPic), with the *M. terrapin* silhouette credited to Shelby Truckenbrod.

Species tree estimation based on 6,000 aligned single-copy orthologs from the *Vertebrate dataset* recovered the same topology for Testudines as reported by Thomson et al. (2021). Given that our calibration points followed those used in Joyce et al. (2013) and Wang et al. (2013), the estimated divergence times were also consistent with those of Thomson et al. (2021) across all nodes of the tree (Fig. 1).

**Figure 2:**
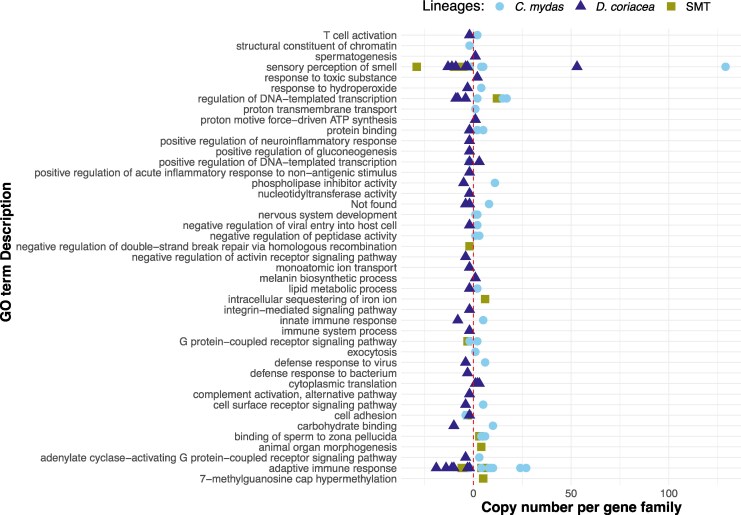
Marine turtles present rapid evolution in genes from olfactory perception and the immune system. GO terms associated with gene families with rapid evolution (*P* > 0.01) for marine turtles, estimated with the *Vertebrate dataset*. Each point represents a gene family (orthogroup). Olive squares represent gene families for SMT, light blue circles for *Chelonia mydas*, and purple triangles for *Dermochelys coriacea*. Red line represents no change, points to the right of this line represent expansions while points to the left represent contractions.

Using the *Vertebrate dataset* and the 19,701 orthogroups identified by OrthoFinder2, CAFE5 modeled changes in gene family size across 14,452 of these orthogroups. Elevated levels of gene family contraction, relative to ancestral nodes, were observed along the stem lineages of Testudines, the stem lineage of marine turtles (SMT), and *P. sinensis* (Fig. 1). On the SMT branch, gene families showing significant signatures of rapid evolution (*P* < 0.01) included contractions in orthogroups related to immune system function and olfactory reception, and expansions in those associated with transcriptional regulation, iron storage, cytoskeletal motor activity, and fertilization (Fig. 2). In *C. mydas*, expansions were observed in gene families annotated as olfactory receptors, zinc finger proteins, immunoglobulins, and other immune-related proteins. In contrast, *D. coriacea* exhibited a predominant pattern of gene family contraction, with the notable exception of an expansion in olfactory receptor family 52 (Fig. 2; Table S8). The major GO terms associated with rapidly evolving gene families in marine turtles are summarized in Fig. 2 and detailed in Table S8. Turtle silhouettes retrieved from https://www.phylopic.org/.

### Twisst: topology weighting

To explore potential phylogenetic signals associated with habitat, we performed topology weighting on 11,262 gene trees derived from single-copy ortholog alignments across seven turtle species representing distinct ecological niches. This analysis revealed 15 distinct topologies, with three predominant patterns differing mainly in the placement of Chelonioidea and Trionychoidea (Figure S3). The most frequently supported topology was consistent with the currently accepted Testudine phylogeny (Thomson et al. 2021). In contrast, topologies that grouped marine turtles (Chelonioidea) with the saltwater-adapted *M. terrapin* were rare, represented in only 62 gene trees (0.05%) and corresponding to topologies “topo3,” “topo10,” and “topo15” (Figure S3).

GO enrichment analysis of genes supporting topologies that grouped all saltwater turtles revealed biological processes potentially related to adaptation to marine and brackish environments. Enriched terms included positive regulation of the mitotic cell cycle (GO:0045931), intestinal epithelial cell differentiation (GO:0060575), positive regulation of vasculature development (GO:1904018), columnar/cuboidal epithelial cell differentiation (GO:0002065), positive regulation of angiogenesis (GO:0045766), gonad development (GO:0008406), and regulation of focal adhesion assembly (GO:0051893) (Table S9). In addition, individual genes involved in oxidative stress response (*RHOA*) and water transport (*AQP8*) also supported these topologies. The full list of enriched GO biological processes is provided in Table S10.

### Positive selection analysis

**Figure 3:**
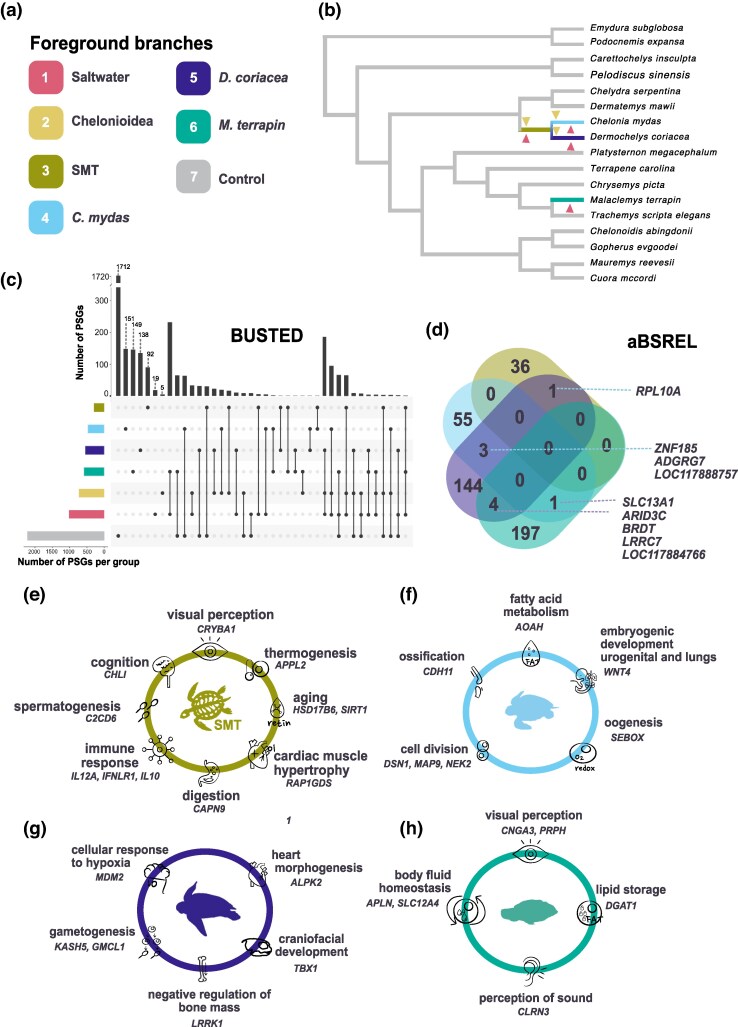
Molecular basis of sea life adaptation in turtles. (a) Different hypotheses tested on the Busted analysis. (b) For hypotheses with more than one foreground branch triangles show which branches were marked. All gray branches were marked for the control hypothesis. (c) Number of PSGs found for each test using BUSTED and their overlap across tests. (d) Number of shared and exclusive PSGs per lineage, identified by aBSREL. Dashed lines point to PSGs associated with group intersections. Functions and genes potentially involved in marine adaptation are highlighted for the SMT (e), *Chelonia mydas* (f), *Dermochelys coriacea* (g), and *M. terrapin* (h) among the exclusive PSGs identified by aBSREL for each lineage. Color scheme per test (BUSTED) and lineage (abSREL): pink for Saltwater branches, yellow for marine branches, olive for SMT, light blue for *C. mydas*, purple for *D. coriacea*, and teal for *M. terrapin*.

Using BUSTED, we tested seven hypotheses by assigning different sets of branches in the turtle phylogeny as the targets for detecting episodic positive selection (Fig. 3a and b). We detected evidence of episodic, gene-wide diversifying selection in 1,022 genes across all four saltwater lineages (the branches subtending each of the two marine turtles, their common ancestor, and the branch subtending to *M. terrapin*). Specifically, we identified: 1,022 positively selected genes (PSGs) in the combined saltwater lineage (SMT + *C. mydas* + *D. coriacea* + *M. terrapin*) in test 1; 735 PSGs in Chelonioidea (SMT + *C. mydas* + *D. coriacea*) in test 2; 300 in SMT (test 3); 472 in *C. mydas* (test 4); 546 in *D. coriacea* (test 5); and 585 in *M. terrapin* (test 6). As a reference, test 7 (the control test) recovered 2,216 PSGs (see Materials and Methods for test definitions). All PSGs and their false discovery rate (FDR) corrected *P*-values (<0.05) are listed in Table S11.

These analyses indicate that at least one site in each of these genes has experienced diversifying positive selection in one or more of the designated foreground branches. Genes uniquely identified in each test, as well as those shared across multiple tests, are detailed in Table S12. Figure 3c illustrates the extent of overlap among tests and the number of PSGs identified in each test. Table 1 highlights PSGs shared between *M. terrapin* and at least one marine turtle lineage. The corresponding likelihood ratio test (LRT) statistics and adjusted *P*-values for each gene are provided in Table S13.

**Table 1:** Genes with sign of diversifying positive selection shared by at least one marine turtle lineage and *M. terrapin* estimated with BUSTED. Pathways were retrieved from the GO Biological Process 2023 database from Enrichr analysis. Positive selected genes (PSG). LRT > 3.84 and *P*-values <0.05. Total gene count for each test in parentheses.

Test	Gene count	PSG
Saltwater, Marine, *D. coriacea*, *M. terrapin*	(11)	TRPA1,SLC16A4,PTPRU,UBQLN4,CCDC170,LOC117884766, FBXO21, NAV1, LRRC7, LOC117869079, LOC117875213
Saltwater, *D. coriacea*, *M. terrapin*	(5)	SMG1, TMBIM6, BRDT, APLP2, HEMGN,
Saltwater, Marine, *M. terrapin*	(5)	LUZP1, XRCC1, MAGI3, PCYOX1, OXNAD1
Saltwater, Marine, *C. mydas*, *M. terrapin*	(4)	SLC13A1, AP4B1, AKR7A2, S100A11,
Saltwater, Marine, SMT, *M. terrapin*	(3)	GPR84, ONECUT3, TNFSF4
Saltwater, SMT, *M. terrapin*	(2)	DEPDC1, IFNLR1
*C. mydas*, *M. terrapin*	(2)	F9, GGPS1
Saltwater, Marine, SMT, *C.mydas*, *M. terrapin*	(1)	CCDC28B
Saltwater, *C.mydas*, *M.terrapin*	(1)	UMODL1
*D. coriacea*, *M. terrapin*	(1)	IL15

Although GO terms related to immune response regulation were found among PSGs shared by the saltwater-adapted species (Table S14), the enrichment analysis using Enrichr did not identify any significantly enriched GO categories after correction for multiple testing. This suggests that while individual genes may show signatures of positive selection, they do not cluster into a specific functional category with statistical significance.

After the exclusion of shared PSGs, the number of exclusive PSGs for each test was 1,712 for the control test, 151 for *C. mydas*, 149 for *M. terrapin*, 138 for *D. coriacea*, 92 for SMT, 19 for the saltwater test, and 5 for the marine test (Fig. 3c). For a complete list of exclusive PSGs retrieved for each test and their corresponding GO terms, see Table S15. Notably, several exclusive PSGs identified in the collective saltwater and marine tests showed evidence of positive selection on at least one foreground branch. This indicates that grouping lineages into a collective foreground increases the statistical power of the BUSTED framework, enabling the detection of subtle or cumulative signals of selection that may remain statistically “silent” when lineages are analyzed individually (Murrell et al. 2015; Kosakovsky Pond et al. 2020). Overall, no gene exhibited strong evidence of gene-wide positive selection across all four saltwater lineages simultaneously using BUSTED.

aBSREL returned evidence for episodic positive selection across several branches throughout the phylogeny (Table S16). A total of 36 exclusive genes with episodic positive selection were found for SMT. Among them, we highlight sirtuin 1 (*SIRT1*), telomeric repeat binding factor 2 (*TERF2*), Interleukin 10 (*IL10*), interferon lambda receptor 1 (*IFNLR1*), tumor necrosis factor ligand superfamily member 4 (*TNFSF4*), and crystallin beta A1 (*CRYBA1*). Figure 3a and Table S17 list the PSGs that are shared among turtle species as well as those that are exclusive to specific lineages. In addition, 55 exclusive PSGs were found for *C. mydas*, 144 for *D. coriacea*, and 197 for *M. terrapin*. Functions associated with PSGs that could be related to marine or saltwater adaptation and that were retrieved by aBSREL for those four branches are shown in Fig. 3e–h. No evidence of positive selection on the same site and the same gene was found among all the marine turtle branches and *M. terrapin*. GO Terms for exclusive PSGs for each marine turtle branch and *M. terrapin* are shown in Tables S18–S21 and REVIGO Summarized GO terms are shown in Figures S4–S7.

Both BUSTED and aBSREL identified numerous genes with signatures of positive selection in the target species and lineages. The fact that certain genes were identified in grouped BUSTED tests but not in all individual aBSREL tests suggests a “dilution” of signal in individual analyses; in these cases, the selective pressure may be present but episodic or distributed across multiple sites, requiring the pooled power of a grouped foreground for detection (Enard et al. 2014). While each method detects partially distinct sets of PSGs, several genes were consistently identified by both approaches. Table 2 presents the PSGs detected by both BUSTED and aBSREL for each lineage, and Table S22 shows the associated GO terms for all PSGs.

**Table 2:** Genes with signs of positive selection retrieved by both methods, BUSTED and aBSREL. *P*-values are corrected for multiple comparisons by FDR with the Bonferroni–Holm procedure.

		BUSTED		aBSREL	
Lineage	Gene	LRT	*P*-value	LRT	*P*-value
*C. mydas*	*ANPEP*	17.6226	0.0012	8.009	0.0226
	*CDC26*	15.5825	0.0035	13.727	0.0034
	*LOC117882921*	14.5889	0.0067	9.896	0.0125
*D. coriacea*	*ADAM23*	17.1863	0.0019	4.833	0.0468
	*AGBL3*	15.7704	0.0032	8.496	0.0135
	*CHD6*	24.3579	0.0000	17.907	0.0003
	*DCTN1*	14.8204	0.0059	5.493	0.0377
	*FAM162A*	26.1404	0.0000	8.460	0.0136
	*KCTD20*	21.3923	0.0002	10.493	0.0067
	*NECAP1*	16.3720	0.0025	7.117	0.0223
	*NUS1*	13.8409	0.0091	9.349	0.0103
	*PRNP*	16.2723	0.0029	5.265	0.0402
	*SCARB1*	20.9003	0.0003	9.936	0.0083
	*SLC45A2*	14.7919	0.0062	14.263	0.0014
	*SULT1B1*	17.3073	0.0014	10.864	0.0058
	*TBX1*	20.4685	0.0003	13.699	0.0018
	*ZAR1*	16.4033	0.0025	7.033	0.0230
*M. terrapin*	*CATSPERG*	26.1641	0.0000	24.332	0.0000
	*CDC37L1*	14.8327	0.0051	11.268	0.0035
	*CPE*	16.0998	0.0032	17.565	0.0003
	*DCAF10*	24.9436	0.0000	9.015	0.0091
	*DLGAP5*	21.4114	0.0002	20.731	0.0001
	*HDHD5*	19.7602	0.0005	18.779	0.0002
	*HSPA9*	13.7747	0.0097	5.136	0.0413
	*IFT140*	43.8018	0.0000	29.960	0.0000
	*KLF15*	18.6872	0.0008	8.002	0.0133
	*LOC117881771*	16.3984	0.0029	14.950	0.0008
	*LRRD1*	26.8415	0.0000	22.262	0.0000
	*NBN*	16.9454	0.0022	14.845	0.0008
	*PLCB2*	21.9194	0.0002	16.842	0.0004
	*SPC25*	26.2774	0.0000	17.728	0.0002
	*SPDYC*	20.4265	0.0004	14.877	0.0008
	*STAT4*	23.0824	0.0001	14.105	0.0011
	*TESK2*	18.7693	0.0009	14.016	0.0011
	*WBP2NL*	14.5733	0.0067	13.561	0.0013
	*YLPM1*	26.1119	0.0000	18.460	0.0002
	*ZBTB40*	18.1715	0.0011	15.713	0.0006
SMT	*RHCE*	15.4259	0.0045	7.354	0.0283

### Historical demography

**Figure 4:**
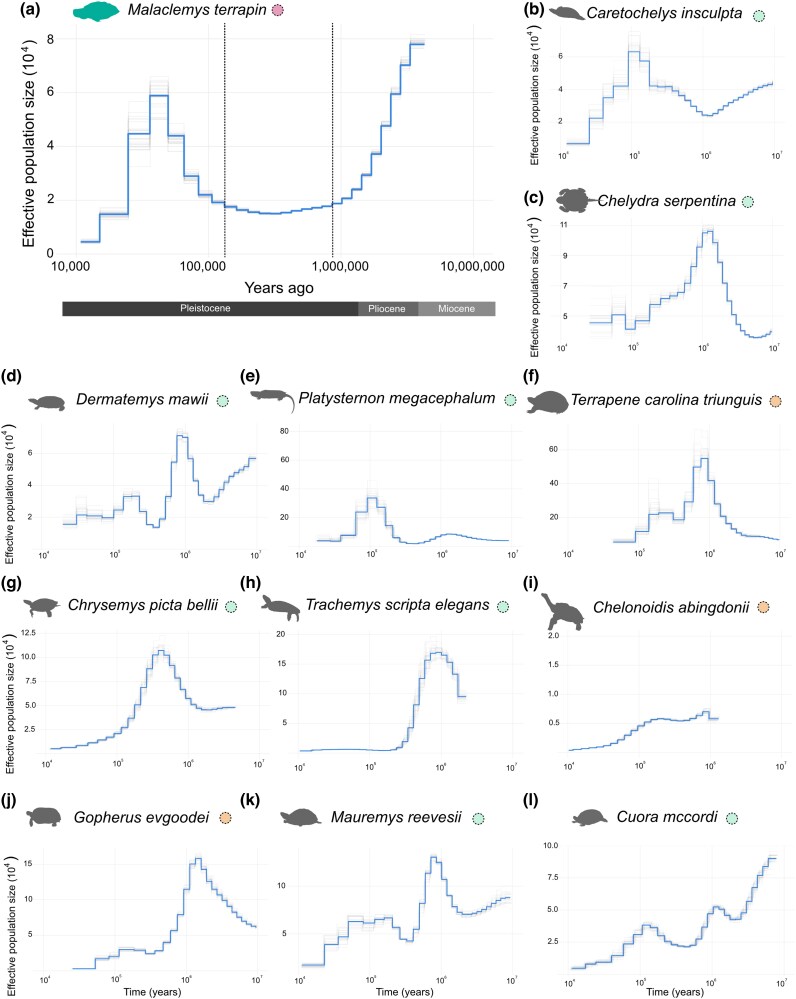
Effective population size (N_e_) inferences for chelonian species. Inferred fluctuations in N_e_ were rescaled assuming different generation times for each species (see Methods) and 1.0 × 10^−8^ per generation mutation rate. Faint lines indicate uncertainty of inferred Ne based on 25 bootstrap replicates. Circles besides species names are color-coded by habitat: freshwater (mint green), saltwater (pink), and terrestrial (orange). (a) *Malaclemys terrapin* showing intense decline followed by stability and a recent increase in population size. Dashed lines indicate the Last Interglacial (Eemian Period, 130,000 to 115,000 years ago) and mid-Pleistocene transition (1.2 to 0.5 mya). (b–l) PSMC estimates for many non-marine chelonians showing great variance in demographic stories.

Given the very large difference in the time that marine turtles and *M. terrapin* have existed as marine taxa, we explored the demographic history of terrapins and compared it to both marine turtles (analysis from (Bentley et al. 2023; Arantes et al. 2025) and to other freshwater and terrestrial species for which reference genomes are available. Using Pairwise Sequentially Markovian Coalescent (PSMC) models, we reconstructed for the first time the demographic histories of 12 chelonian species, uncovering divergent population trajectories over the last several million years (Fig. 4). *Malaclemys terrapin,* with a stem age of roughly 15 million years (Thomson et al. 2021, see Fig. 1), exhibited a maximum effective population size (N_e_) approximately 7 to 9 million years ago (mya), followed by a sustained decline reaching a minimum 200,000 to 300,000 years ago. A secondary increase in N_e_ occurred between 40,000 and 60,000 years ago, followed by a decline toward the present. While this broad “U-shaped” profile mirrors the demographic declines reported for fully marine turtles (Bentley et al. 2023; Arantes et al. 2025), the recovery peak in *M. terrapin* occurred later than those observed in Chelonioidea. The demographic patterns for the non-marine turtles analyzed here were heterogeneous, varied considerably and lacked the synchronized ancient contractions found in the marine-adapted species. Although recent PSMC peaks can occasionally arise from parameter artifacts, we mitigated this risk by following the optimization protocols suggested by Hilgers et al. (2025). Consequently, the distinct trajectory observed for *M. terrapin* likely represents a genuine biological signal, though the terminal decline warrants cautious interpretation, as it may be influenced by population structure (Mazet et al. 2016).

### Evolutionary rates comparison

Evolutionary rates were broadly consistent across the 17 turtle species tested based on 14,898 single-copy orthologs (Figure S8). A distinct gradient in substitution rates was observed among the studied taxa, with the highest median rates occurring in *C. insculpta* and the most constrained pace in *P. megacephalum*. Marine turtles (*C. mydas* and *D. coriacea*) exhibited highly synchronized intermediate rates—potentially reflecting shared life-history constraints—while the *M. terrapin* maintained a similar median but displayed a higher frequency of accelerated outliers compared to the marine species. These results strongly align with the genomic signatures of evolutionary stasis described in previous literature, placing the median rates (approx. 0.00015 substitutions/site/million year) within the established range for turtles as a slowly evolving lineage (Shaffer et al. 2013; Brownstein et al. 2024).

## Discussion

The transition to a marine lifestyle presents significant physiological and behavioral challenges that various amniote lineages have overcome. A key evolutionary question is whether these independent transitions rely on the same underlying molecular mechanisms. We identified a broad set of genetic modifications unique to marine turtle lineages, distinguishing them from terrestrial, freshwater, and estuarine turtles. Gene families showing signs of rapid evolution in marine turtles are concentrated in functions related to immunity, olfactory perception, and lipid metabolism. We also found specific nucleotide changes in the marine lineages tested, potentially linked to marine adaptation that appear to be maintained by natural selection. Notably, several genes under positive selection and historical demographic profile are shared between marine turtles and the saltwater estuarine turtle *M. terrapin*, suggesting repeated molecular responses to saline environments. These patterns also indicate that turtles underwent genomic changes in pathways similar to those altered in other marine vertebrates.

### Marine turtles genomes show rapid evolution in sensory perception gene families

Marine turtles show more gene family contractions than expansions compared to other turtles and vertebrates, particularly in olfactory receptor and immune system families. This pattern was previously observed in genomic studies (Wang et al. 2013; Bentley et al. 2023), and is now confirmed with our broader turtle taxonomic sampling. Gene families related to taste and smell reception, especially olfactory receptors (ORs), are significantly contracted in the marine turtle stem lineage (SMT), reflecting reduced reliance on chemosensation in marine environments. Similar genomic signatures have also been identified in seabirds; for instance, banded penguins exhibit significant contractions in gene families encoding odorant receptors and sodium chloride-channel proteins, suggesting that the loss of specific sensory and physiological pathways is a recurrent feature of the transition to an aquatic lifestyle (León et al. 2024). This trend is consistent with secondary marine colonization in other vertebrates, including mammals and snakes (Zhu et al. 2014; Kishida et al. 2019; Liu et al. 2019; Policarpo et al. 2024).

Turtles generally possess the largest repertoire of intact OR genes among reptiles (Policarpo et al. 2024), a finding supported by early observations of OR expansions in the *C. mydas* draft genome (Wang et al. 2013). By expanding the taxonomic sampling of chelonian species relative to Bentley et al. (2023) to include a diverse range of non-marine and estuarine lineages, we confirm that the marine transition is marked by significant gene turnover. This process began with an ancestral OR contraction in the stem lineage of sea turtles—mirroring the early sensory gene loss seen in cetaceans (Kishida et al. 2015)—followed by subsequent expansions in extant lineages on the OR52 family, which is specialized for detecting hydrophilic compounds. These gains are more pronounced in *C. mydas* than in *D. coriacea*, suggesting a functional shift toward aquatic olfaction that varies in intensity across the marine clade. Although most turtle genomes contain three vomeronasal receptor genes (Policarpo et al. 2024), one VR2 gene (*LOC117883272*) is absent in both marine turtles and *M. terrapin* when compared with the freshwater species *T. scripta elegans*. However, this gene is also lost in *C. insculpta* and *G. evgoodei*, suggesting the pattern is not exclusive to saline environments.

### Turtles inhabiting saltwater exhibit similarities in genes related to sensory perception and ion and water homeostasis

Our analyses revealed PSGs unique to *C. mydas*, *D. coriacea*, and *M. terrapin*, offering insights into trait-specific adaptation. Many of the positively selected genes we identified have known functions consistent with the selective pressures expected in marine habitats. While these associations are supported by functional annotations (eg GO terms) and comparative literature, they remain correlation-based hypotheses, and should be interpreted as such in the absence of direct functional or phenotypic validation. Further studies using transcriptomic, phenotypic, or biochemical approaches will be essential to confirm the mechanistic links between these genetic variants and marine adaptation. Nevertheless, PSGs identified at macroevolutionary scales represent candidates for investigating genotype–phenotype relationships and lay a foundation for future experimental and population-level research. Below, we detail our candidate marine-associated PSGs and their possible role in the adaptation of marine and salt-water turtles.

Marine turtles and *M. terrapin* share signatures of gene-wide positive selection in genes related to sensory perception (*TRPA1*), kidney and intestine ion transport (*SLC13A1*), and bladder disease (*DEPDC1)*. *TRPA1* was previously reported to regulate the behavioral response of starfish larvae to environmental temperature changes (Saito et al. 2017) and changes maintained by positive selection on *TRPA1* in our study points to its relevance to marine adaptations. Positive selection signatures in genes related to sodium/sulfate reabsorption in the kidney and small intestine (*SLC13A1*) (Lee et al. 2000) and bladder cell disorder (*DEPDC1*) (Harada et al. 2010) could also reflect adaptations that enabled turtles to successfully colonize saltwater environments. Another gene for which saltwater turtle species show signals of positive selection is *S100A11*. The protein encoded by this gene is a regulator of epidermal keratinocytes, and a potential different structure in saltwater turtle species may play a role in skin impermeability to saltwater (Sakaguchi and Huh 2011).

We identified gene-wide evidence of positive selection in *M. terrapin* for genes associated with GO terms previously linked to accelerated evolution in marine vertebrates, including sensory perception and osmoregulation. These patterns, also observed in at least one marine turtle lineage, suggest that similar selective pressures may act on the same genes, albeit at different sites within those genes. The absence of identical amino acid substitutions in *M. terrapin* and marine turtles may be expected, given their distant evolutionary relationships and the general rule that precise molecular convergence is most likely among closely related species that share similar genomic contexts (Bridgham 2016; Storz 2016; Agrawal 2017). In contrast, more distantly related taxa often exhibit convergence at broader functional levels rather than at specific sites in the same gene. Although striking examples of site-level convergence have been reported between highly divergent lineages—such as the independent evolution of specialized limbs in giant and red pandas (Hu et al. 2017) or spectral sensitivity in diverse bony fish families (Hill et al. 2019)—these typically involve cases where strong ecological selection acts on a highly constrained set of functional residues (Hughes 2007). Comparative studies across diverse taxa increasingly suggest that molecular convergence more commonly involves different substitutions in genes with similar functions, rather than identical changes at the same sites (Zhou et al. 2015; Morales et al. 2024). This highlights the role of phylogenetic constraints and opportunities, and the importance of evolutionary scale in interpreting patterns of convergence. Hughes (2007) further emphasized that repeated site-specific substitutions are exceptional and often depend on the specialized functional roles of the proteins involved. Finally, differences in habitat between marine turtles and the estuarine *M. terrapin* may also shape the strength and direction of selective pressures. Collectively, these observations underscore the complexity of repeated evolution and the importance of considering phylogenetic relatedness, environmental and ecological similarity, and genomic architecture when evaluating evolutionary repeatability.

We also observed gene tree topologies that deviate from the species tree, including for *AQP8*, a gene involved in water transport (Yang et al. 2005a). The *AQP8* gene tree unexpectedly groups *M. terrapin* with Chelonioidea rather than its sister clade, Testudinoidea. This phylogenetic placement likely reflects similar sequence modifications between *M. terrapin* and marine turtles. Since our screens found no evidence of diversifying positive selection in this gene, these shared features may have independently evolved in both of these distantly related lineages due to stabilizing selection or relaxation of purifying selection, since it was not detected by screens for signatures of diversifying positive selection. *AQP8* has been proposed to be a potentially important water transporter in the gastrointestinal tract of mammals (Yang et al. 2005a).

### Specific signatures of selection for the two sea and saltwater turtle lineages

#### Diving physiology

Among marine turtles, *D. coriacea* are capable of the deepest dives (Lutz and Lutcavage 2017). Several morphological and physiological adaptations have been proposed as facilitators of deep diving in *D. coriacea*. These include a greater oxygen storage capacity in blood and muscle tissues compared to the lungs (Lutcavage et al. 1992), high cardiac frequencies at the surface (Southwood et al. 1999), and a “gigantothermy” strategy (Paladino et al. 1990) that maintains lower body temperatures than that of endotherms, thereby reducing the risk of nitrogen bubble formation during dives (Fossette et al. 2010). Furthermore, Fossette et al. (2010) noted that *D. coriacea* diving patterns expose individuals to high energetic costs. In our analysis, we found PSGs associated with energy generation (*TFAM*) and oxidative stress response (*MDM2, IFNG, PRDX5, STOX1, IPCEF1*) in *D. coriacea*, suggesting that modifications in these genes may be involved in the necessary trade-offs for the evolution of deep diving capacity. Modifications in genes from nitric oxide metabolism were also found on the polar bear genome and are associated with the diving capacity of this species since nitric oxide is assumed to play a role in coordinating tissue energy demand (Welch et al. 2014).

*Chelonia mydas* also exhibits several PSGs for hypoxia adaptation, including *ZEB1* and *NOTCH1*. *ZEB1* is linked to hypoxia adaptation in Tibetan sheep (Liu et al. 2020), while *NOTCH1* is crucial for improved hypoxia tolerance in high-altitude human populations (Bigham et al. 2009; Xin et al. 2020). For *C. mydas*, positive selection signals were also found for *F2RL1*, which is associated with vascular smooth muscle relaxation, blood vessel dilation, increased blood flow, and lowered blood pressure in other species. This gene shows adaptive signals in Tibetan and Dahe pigs, both of which suggest a role in high-altitude adaptation (Dong et al. 2014). Additionally, *PSTK*, a phosphorylase kinase protecting against oxidative stress (Zheng et al. 2015), is under selection in *C. mydas*.

#### Osmoregulation

Marine turtles and *M. terrapin* use lachrymal salt glands to excrete excess salt (Bentley et al. 1967; Lutz 2017). In *M. terrapin*, we found selection on *SLC12A4*, a K+/Cl− transporter that is also under selection in euryhaline fishes (Zhang et al. 2020). Our findings provide preliminary insight into the genetic mechanisms of osmoregulation in the saltwater turtle *M. terrapin*, which may underlie its adaptive evolution to salinity tolerance. Examination of these genes in related freshwater emydid turtles would be particularly interesting as a future research direction.

#### Immune system

PSGs related to DNA repair, tissue regeneration, and immune response were identified in *C. mydas*. This species also shows an expanded repertoire of immune system genes relative to *D. coriacea* (Bentley et al. 2023), suggesting accelerated immune evolution. Such changes may reflect adaptation to more coastal marine habitats, where pathogen exposure is higher (Newell 2013), and where *C. mydas* experiences frequent infections, including Chelonid Herpesvirus 5 (Jones et al. 2016; Page-Karjian 2019). Indeed, evolutionary acceleration in immunity-related genes has been linked to species from different aquatic environments, including turtles in freshwater habitats compared to the land relatives (Liu et al. 2022). However, convergent changes in amino acids of genes related to the DNA repair process were found between the green turtle and sea snakes, suggesting that this process evolves under strong selection in these independently evolved marine environment specialists (Peng et al. 2020). Selection on *IFNG* in *D. coriacea*, also found in long-lived bats (Moreno Santillán et al. 2021), highlights a potential link between immune function and longevity. Further research across chelonid species is needed to determine whether rapid immune evolution is unique to *C. mydas* or more widespread among coastal marine turtles.

#### Muscle-related genes

We identified two muscle-related genes under positive selection in *D. coriacea*: *MYH9* and *MSS51*. Both genes have functional relevance in skeletal muscle performance and show interesting parallels with adaptations reported in marine mammals. *MYH9*, part of the myosin gene family, has a similar evolutionary signal to *MYH7*, which has been repeatedly targeted by selection in independent marine mammal lineages (Foote et al. 2015; Chikina et al. 2016). MSS51, predominantly expressed in fast glycolytic muscle fibers, was also found to be under selection. Inactivation of MSS51 shifts energy metabolism toward fatty acid oxidation, and notably, this gene was lost in the cetacean stem lineage—consistent with their reliance on lipid-based muscle metabolism and high intramuscular fat content (Huelsmann et al. 2019).

#### Diet and visual perception

We found strong evidence of positive selection in genes related to diet and vision in both marine and estuarine turtles. In *C. mydas*, PSGs included *TBX1* and *SELENOT*, potentially reflecting adaptations to dietary variation, particularly in selenium availability. Selenium-related genes are known to evolve under different selective pressures depending on environmental and nutritional contexts (Sarangi et al. 2018), suggesting that similar forces may shape Se-related gene evolution in some marine turtles.

In *M. terrapin*, selection on *CNGA3*, a gene involved in phototransduction, may indicate visual adaptation to estuarine habitats, which are often characterized by fluctuating turbidity and low-light conditions (Lunt and Smee 2020). Vision is likely a key sensory modality in such environments. Comparable selection on *CNGB1* in penguins has been linked to enhanced visual sensitivity for underwater foraging (Cole et al. 2022), supporting the possibility of similar selective pressures acting on *M. terrapin*.

Future genomic research focusing on diet and visual perception genes in marine turtles may reveal deeper insights into their ecological adaptations. Comparative studies investigating selenium-related and photoreceptor genes across different turtle lineages could provide a broader understanding of the evolutionary processes that shape their dietary flexibility and visual adaptations.

### Adaptation of similar biological processes drives marine adaptation of turtles, snakes, birds, and mammals

In the SMTs, we identified positive selection acting on genes involved in lipid metabolism and storage, cellular energy production, immunity, cold acclimation, digestion, and sensory perception. These functions are crucial for coping with the physiological demands of a marine lifestyle, including long-distance migrations, deep or prolonged diving, thermal variability, and limited access to freshwater. The patterns observed in SMT parallel those found in marine mammals, birds, and snakes following secondary colonization of marine environments (McGowen et al. 2012; Peng et al. 2020; Cole et al. 2022), suggesting that similar selective pressures have shaped convergent molecular responses in all of these lineages.

Adaptations in lipid metabolism in SMT may be particularly important for sustaining energy and producing metabolic water during long migrations, extended fasting periods during nesting or developmental stages, and prolonged submergence (Ortiz 2001; Castellini and Mellish 2015). The positive selection we observed in Sirtuin 1 (*SIRT1*) in SMT, a gene associated with longevity and deep-diving capacity in mammals (Junker and Gossmann 2021; Piotrowski et al. 2021), is consistent with the long lifespans and repeated hypoxic stress experienced by marine turtles. Similarly, the signatures of selection detected along the SMT lineage on telomeric repeat binding factor 2 (*TERF2*), a gene involved in telomere maintenance in humans (Ye et al. 2010), may reflect adaptations related to longevity and cellular maintenance under oxidative and thermal stress in turtles. Evidence of changes potentially maintained by natural selection on this gene was previously reported for many species with increased longevity, including the naked mole-rat (Kim et al. 2011) and bats (Foley et al. 2018), gigantism, like the capybara (Herrera-Álvarez et al. 2021) and species with remarkable phenotypic specializations to new habitats, like mole-rats (Davies et al. 2015).

We also detected selection in SMT on *IFNLR1*, a gene linked to adaptation to hypoxic conditions in mammals (Guang-Xin et al. 2020), which is likely important for SMT due to the low-oxygen conditions they experience during extended dives. The signal of positive selection we found on *CRYBA1* in the SMT may reflect adaptive tuning of visual function to low-light underwater environments, in line with previous associations of this gene with photic adaptation in crepuscular marsupials (Tian et al. 2022). Supporting this, *CRY* genes also show adaptive evolution in other aquatic vertebrates like Iberian *Squalius* freshwater fish, where temperature appears to act as a selective force (Moreno et al. 2021). Given the wide thermal ranges encountered by SMT during oceanic migrations and life stages, circadian and photoreceptive gene adaptation may be essential for synchronizing physiology and behavior to environmental cues.

Our results show that positive selection has acted on the stem lineage of marine turtles in some of the same genes, such as *SIRT1* and *IFNLR1*, or in similar biological processes as those targeted in marine mammals (Xu et al. 2012; Montgomery et al. 2014; Junker and Gossmann 2021; Piotrowski et al. 2021). However, it remains unclear whether these adaptations involve the same nucleotide changes across different secondarily marine vertebrate lineages, especially when signals of selection are detected in the exact same genes. Comparative sequence-level analyses of these genes will be key to uncovering the molecular patterns underlying repeated evolution in secondarily marine vertebrates.

### Population fluctuations in marine-adapted lineages

Our demographic reconstructions reveal that while *M. terrapin* shares a general history of climate-linked declines with fully marine turtles, it exhibits a distinct temporal shift in its recovery and expansion. The ancient population peak (∼6 mya) and subsequent decline in *M. terrapin* mirror the trends seen in marine turtles, including *C. mydas* and *D. coriacea* (Arantes et al. 2025), and may reflect a shared sensitivity to Pliocene-Pleistocene cooling, a period marked by profound oceanographic temperature shifts and the intensification of Northern Hemisphere Glaciation (Bartoli et al. 2005). However, a key distinction emerges in the mid-Pleistocene: while many marine turtles underwent synchronous expansions between 500 kya and 1.2 mya (Bentley et al. 2023; Arantes et al. 2025), *M. terrapin* remained in a prolonged population bottleneck. This is consistent with the interpretation that the estuarine marshes required by *M. terrapin* were more severely impacted by sea-level regressions than the pelagic habitats of marine turtles. The secondary spike in population size observed around 40,000 to 60,000 years ago for *M. terrapin* aligns with the milder periods preceding the Last Glacial Maximum. While we optimized our PSMC parameters to split the initial time intervals to reduce the likelihood of parameter-induced artifacts (Hilgers et al. 2025), we acknowledge that terminal declines within the last 100,000 years can be influenced by population structure. Because PSMC assumes a single panmictic population, the presence of distinct subpopulations can lead to an overestimation of recent declines (Mazet et al. 2016). Nevertheless, the broad synchrony of these shifts across marine adapted lineages highlights a shared sensitivity to fluctuating sea levels.

### Prospects for marine turtle marine adaptation genomics

Our study represents an important first step toward understanding the molecular signatures potentially associated with turtles’ successful re-colonization of marine environments. Here, we focus on marine-differentiated genes that likely have resulted from adaptive evolution, exploring the presence of positive selection signs in gene sequences from marine turtles. However, marine turtle genomic uniqueness could also be a result of the relaxation of selection in response to the environment, as evidenced by Chikina et al. (2016) in marine mammals, and such shifts in the stringency of natural selection should be also investigated in marine turtle genes in the future.

Distinguishing between the genomic footprints of natural selection and the artifacts of demographic fluctuations remains a central challenge in evolutionary biology (Tajima 1989; Ellegren 2008). While the selection signatures identified in this study align with ecologically relevant traits, the confounding influence of demographic history cannot be entirely dismissed, particularly for lineages such as *M. terrapin* that experienced severe and prolonged bottlenecks. Distinguishing whether genomic variations on those lineages are genome-wide (demographic) or localized (selective) remains an avenue for future research and will require population-level genomic data (Kim and Stephan 2002). By identifying specific genomic regions of interest, our study provides a foundation for future investigations to definitively characterize these variants as adaptive signatures or demographic artifacts. Nevertheless, the identification of candidate genes that show repeated signals across independent lineages, each subjected to similar marine environmental pressures, provides strong circumstantial evidence for their adaptive nature, as demographic noise is less likely to produce such consistent patterns across divergent clades.

Although all turtle superfamilies are currently represented by at least one species in genome databases, the taxonomic distribution of available genomes remains uneven. At the time of this study, only 26 of the 364 described turtle species had genome assemblies available (TTWG 2025), and while 25 of these were assembled to the chromosome level, more than half had been released only in the past two years. Importantly, some sub-clades remain underrepresented or are only represented by a single species, limiting robust comparative analyses within those groups. Furthermore, many genomes lack functional annotations, underscoring the need for improved standardization and broader taxonomic coverage in future turtle genome sequencing efforts.

Finally, a comparative genomic analysis of the genomes of the five other marine turtle species is needed to make a comprehensive evolutionary analysis of the characters identified here. In addition, with these genomes available, investigating types of genetic variation other than point mutations in coding regions that enable adaptation (i.e. regulatory changes, inversions, gene duplications, repetitive regions) is important to clarify the sources of adaptive genetic variation in this group.

Our investigation of high-quality genomes across diverse chelonian lineages provides insights into the genomic evolution of marine-adapted turtles. By analyzing coding regions in the anciently diverged *C. mydas* and *D. coriacea* alongside the more recent estuarine specialist, *M. terrapin*, we document the varied evolutionary strategies that facilitate the transition from land to sea. We contextualize these findings through a comparative framework, highlighting shared candidate molecular solutions to oceanic life observed across vertebrate lineages. Furthermore, our integration of demographic histories reveals how the timing of population fluctuations and habitat specialization may have helped shape modern genomic diversity, distinguishing the relatively ancient radiations of marine turtles from the recent marine invasion of *M. terrapin*. Collectively, these results provide a robust resource for hypothesis-driven research into turtle evolution and reveal potential cases of repeated molecular adaptation at the gene level. Future work should expand upon this framework by including additional marine turtle species and exploring adaptive variation in other genomic features such as structural variants and repetitive elements. These efforts will advance our understanding of the genomic basis underlying the unique evolutionary trajectories of these iconic, endangered marine vertebrates.

## Material and methods

### Genomic data selection and quality assessment

We downloaded genome assemblies and raw sequencing data for 26 turtle species from NCBI, selecting the most recent versions available at the time of the analysis (Table S1). We performed an assembly quality assessment, using the GEP (https://git.imp.fu-berlin.de/begendiv/gep; Sullivan et al. 2025), which reports key metrics including contiguity statistics, assembly Benchmarking Universal Single-Copy Orthologs (BUSCO) scores (Simão et al. 2015), QV scores, K-mer completeness (Rhie et al. 2020), GenomeScope profiles (Ranallo-Benavidez et al. 2020), and Hi-C contact maps. To remove spurious duplications such as haplotigs and contig overlaps, we applied Purge_dups v1.2.5 (Guan et al. 2020), with the exception of a few assemblies that did not require purging based on copy number spectrum evaluation using GEP (Table S1).

Based on these evaluations, we selected a reduced dataset of 17 turtle genomes that maximized phylogenetic diversity, assembly contiguity, and annotation completeness, including only assemblies with more than 90% complete single-copy orthologs, when more than one assembly was available for the same species. For *C. serpentina*, two different versions of the genome were available. We evaluated both genomes (GCA_007922165.1 and GCA_018859375.1) independently and selected for analysis the one with higher QV after the evaluation of GEP. The selected assemblies represent all major turtle superfamilies and cover a wide ecological range, including marine, freshwater, terrestrial, and estuarine species. Assembly levels range from contig- and scaffold-level to chromosome-level, with 11 genomes assembled to chromosome scale (Table S1). We modeled and masked repeats in all 17 turtle assemblies using RepeatModeler and RepeatMasker, respectively (Tarailo-Graovac and Chen 2009; Flynn et al. 2020).

A detailed summary of species, taxonomic classification, accession numbers, assembly level, and references is provided in Table S7. Several assemblies were produced by the McDonnell Genome Institute at Washington University, while others are linked to published studies, including those for *C. mydas* and *D. coriacea* (Bentley et al. 2023), *C. picta* (Shaffer et al. 2013), *T. scripta elegans* (Brian Simison et al. 2020), *M. reevesii* (Liu et al. 2022), and the big-headed turtle (*Platysternon megacephalum*) (Cao et al. 2019).

### Genome annotation and annotation assessment

To ensure reliable gene content and comparative analyses, we evaluated and supplemented gene annotations across our turtle genome dataset. At the time of this analysis, only eight turtle genomes had publicly available annotations across major databases, including NCBI, European Nucleotide Archive, and other public or species-specific repositories. As we needed to include the highest possible number of representative turtle species in our analysis we projected annotations of protein-coding genes from *T. scripta elegans* to a dataset of 16 turtle genomes using the TOGA (https://github.com/hillerlab/TOGA) (Kirilenko et al. 2023). We chose *T. scripta elegans* as an annotation reference since it was the turtle assembly with the best annotation at the time. Annotation completeness was assessed using BUSCO analysis under three scenarios: (i) based directly on the assembly sequences as part of GEP, (ii) using predicted proteomes from available NCBI annotations, and (iii) using proteomes from TOGA-generated annotations.

TOGA was used to assess the intactness of genes and transcripts. TOGA identifies gene-inactivating mutations such as premature stop codons, frameshifts, and splice site disruptions compared to the reference species: *T. scripta elegans* while accounting for potential confounding factors related to genome assembly quality (Kirilenko et al. 2023). Genes are classified into distinct categories: “intact,” “partially intact,” “clearly lost,” “uncertain loss,” “missing,” or “partial & missing,” based on the presence of mutations and the genomic context (eg scaffold ends or gaps). Genes classified as “clearly lost” are those with strong evidence of inactivation and no indications of assembly or alignment artifacts. Also, TOGA recognizes that a reference gene may be represented by multiple isoforms and identifies when one isoform is lost for a specific gene (transcript lost) or when all isoforms were lost for that gene (gene lost). We relied on this conservative classification to report gene losses, avoiding overinterpretation of absences due to annotation limitations or assembly incompleteness.

### Dataset design and orthology inference

We used two complementary datasets tailored to the specific requirements of each analysis (see Table S7). For gene family evolution and phylogenetic inference, we focused on the subset of eight turtle genomes (the *Vertebrate dataset*) with high-quality annotations derived from species-specific transcriptomic data available through NCBI RefSeq and Ensembl (Thibaud-Nissen et al. 2013). These annotations, informed by lineage-specific RNA data, offer improved gene model accuracy (Li et al. 2011; Rhie et al. 2021; Kirilenko et al. 2023; Vuruputoor et al. 2023). Their completeness was supported by BUSCO scores, which were consistently higher for this subset compared to other annotation strategies (see Results, Tables S1, S2 and Figure S2). For the *Vertebrate dataset*, we used Orthofinder v2.5.4 (Emms and Kelly 2015; Emms and Kelly 2019) to infer orthogroups based on all-vs-all sequence similarity and gene tree reconciliation. Protein sequences were extracted from assembly FASTA files using the GFFreads tool (Pertea and Pertea 2020) based on NCBI annotation coordinates.

To maximize taxonomic and ecological representation for analyses of gene tree discordance and positive selection, we used the expanded dataset (*Turtle dataset*) of 17 TOGA annotated turtle genomes. Besides annotation, TOGA's approach extends orthology inference beyond coding regions by leveraging alignments of intronic and intergenic regions, improving sensitivity and detection of orthologs even in partially annotated or fragmented genomes. In addition to recovering high-confidence orthologous loci, TOGA classifies gene status, detects gene duplications or inactivations, and generates codon and protein alignments. This made it particularly well-suited for broad phylogenetic comparisons across Testudines, enabling the inclusion of additional species with otherwise limited annotation resources. For the *Turtle dataset*, used in gene tree topology and selection analyses, we aligned each target genome to the *T. scripta elegans* reference using the same workflow as in (Blumer et al. 2022). We retained the longest isoform of each one-to-one ortholog that was present in at least 80% of the species. When multiple isoforms of equal length were available, we selected the one that was present and intact in the greatest number of genomes. Codon-aware multiple sequence alignments were then generated using MACSE v2.0 (Ranwez et al. 2018), and alignments were filtered using HmmCleaner (Di Franco et al. 2019). Genes classified by TOGA as lost were excluded from downstream analyses.

Together, these procedures ensured consistent and high-confidence orthology inference across species. To minimize the impact of reference-based annotation, we used the Vertebrate dataset for gene family analyses. For selection scans (*Turtle dataset*), we reduced the impact of missing data by filtering for broad species representation and excluding genes classified as lost, thereby improving the robustness of downstream comparative genomic analyses.

### Phylogenetic inferences

To generate a species tree, we aligned single-copy orthologs obtained by Orthofinder for the *Vertebrate dataset* using MAFFT v6.864b (Katoh and Standley 2013). We removed poorly aligned regions from a multiple sequence alignment using trimAl v1.4 (Capella-Gutiérrez et al. 2009) with the “automated1” algorithm. Then, we concatenated all single-copy trimmed alignments into a super-matrix using geneSticher.py (https://github.com/ballesterus/Utensils/blob/master/geneStitcher.py). Finally, we generated a phylogenetic maximum likelihood tree using IQTREE v 2.1.4 (Minh et al. 2020), with 1,000 bootstrap replicates and considering each copy orthogroup as a partition. The species tree was rooted with the Western clawed frog (*Xenopus tropicalis*). We calibrated the tree using posterior age estimates based on fossil calibrations from Joyce et al. (2013) and Wang et al. (2013) (Table S23). Their estimations were used to convert branch length estimation to absolute divergence dates using r8s (Sanderson 2003). Also, we generated maximum-likelihood gene trees using IQTREE v 2.1.4 (Minh et al. 2020) with automatic selection of nucleotide substitution models for each codon-aligned single-copy ortholog of the *Turtle dataset*.

### Gene family evolution

To assess gene family expansions and contractions in marine turtles, we employed Computational Analysis of Gene Family Evolution v5 (CAFE5) (Mendes et al. 2020). CAFE5 utilizes gene family sizes, incorporating phylogenetic history to identify instances of rapid expansions and/or contractions across all branches in a phylogeny. Prior to this analysis, the assignment of proteins from each genome to orthologous groups representing gene families is essential. Given the enhanced precision of lineage-specific gene annotation, the analysis of gene family dynamics was conducted using the *Vertebrate dataset*, as previously stated. The CAFE5 analysis was performed on ultrametric phylogenies generated in the preceding step, employing default gamma rate categories and lambda estimations.

### Twisst: topology weighting

To investigate gene sequence similarity among turtles inhabiting saltwater, we applied Twisst (Topology Weighting by an Iterative Sampling of Sub-Trees) (Martin and Van Belleghem 2017). This software determines weightings through iterative sampling of sub-trees from the full tree, assessing their topology based on the percentage of occurrences for specific topologies. Due to the optimal performance of the method with a maximum of five groups (Martin and Van Belleghem 2017), we categorized seven turtle species into five groups, based on their superfamilies (Table S7). To include species from different habitats, we used single-copy orthologous alignments from TOGA projections for the marine species *C. mydas* and *D. coriacea* (Chelonioidea), freshwater species *P. sinensis* (Tryonichoidea), *C. serpentina* (Chelydroidea), *M. reevesii*, and terrestrial species *C. abingdonii* (henceforth grouped as Testudinoidea2), along with the saltwater species *M. terrapin* (henceforth labeled as Testudinoidea1). Only single-copy orthologous alignments that contain all the seven species mentioned above were used in this analysis. For this, we filtered the *Turtle dataset* (14,897 alignments) keeping a dataset containing 11,262 alignments. Gene trees employed in this analysis were generated as previously described in the phylogenetic inferences section.

### Natural selection analysis

To access the role of gene-wide episodic positive selection on the genomes of the marine turtle species, we applied the Branch-Site Unrestricted Statistical Test for Episodic Diversification (BUSTED) (Murrell et al. 2015) method from the HyPhy package (Pond et al. 2005). The strength of selection is defined by the ω ratio (dN/dS), in which dS is the rate of synonymous substitutions (ie, silent mutations that do not change the amino acid) and dN represents the rate of non-synonymous substitutions (which confer an amino acid substitution). In the absence of selection, dN and dS should be equal, so ω = 1. Purifying natural selection prevents amino acid substitution, so dN will be less than dS, and ω < 1, while positive directional selection, results in an accelerated amino acid substitution rate and ω > 1. In the BUSTED test, tree branches are split into the foreground (lineage to be tested for positive selection) and background (all other branches assumed not to be under selection). The branches to be tested are specified a priori. Codon sites are split according to their classes based on ω value, but the ω > 1 class is only allowed in foreground branches (Zhang et al. 2005). Then an LRT is employed to compare the alternative (ω > 1 allowed) and the null model (ω = 1) (Zhang et al. 2005; Z. Yang et al. 2005b).

Since the LRT in BUSTED gains sensitivity as the number of foreground branches increases—provided they share similar selection patterns—we tested for positive selection by grouping target species as well as analyzing them individually. This dual approach allows for the detection of positive selection even when the signal is weak within a single lineage. Using codon alignments of one-to-one orthologs from the *Turtle dataset*, we ran seven BUSTED analyses, selecting as foreground branches (i) all four saltwater lineages, including the SMT, *C. mydas*, *D. coriacea,* and *M. terrapin* (ii) the two marine turtle species and SMT, (iii) only SMT, (iv) the *C. mydas* branch exclusively, (v) the *D. coriacea* branch exclusively, (vi) the *M. terrapin* branch exclusively, and (vii) a control test, marking as foreground all other turtle lineages and intermediary branches, with exception of the saltwater turtles. BUSTED determines whether positive selection happened anywhere in the foreground branches, assuming background branches do not experience episodic selection. Therefore, test 7 was conducted to check whether the genes found under positive selection in saltwater species are not also under positive selection in any other turtle species of the tree. The resulting *P*-values for BUSTED have been corrected for FDR using the p.adjust() function in R v3.6.2, with the Bonferroni method as the correction approach (RCoreTeam 2023).

We also used the aBSREL model (Smith et al. 2015) implemented in the Hyphy package (v2.3.11) to identify genes that have experienced branch-speciﬁc episodic selection during the evolution of marine turtles and *M. terrapin*. All branches in the turtle tree were labeled as test branches. *P*-values from individual tests of different branches in the phylogenetic tree were corrected for multiple comparisons by the Bonferroni–Holm procedure to control family-wise false-positive rates (Smith et al. 2015; Spielman et al. 2019).

For both HyPhy algorithms, we conducted tests using species trees. Because the *Turtle dataset* is incomplete for some genes, containing cleaned multiple sequence alignments with at least 80% of the expected turtle species, we pruned the species tree for certain genes, generating a version of the species tree that includes only the species present in each alignment. Tree pruning was performed using the tree_doctor utility from the PHAST package (Hubisz et al. 2011) to create adapted species trees for each alignment. First, ancestral nodes were renamed based on their descendant nodes, and specific nodes were assigned taxonomic names (eg “Pleurodira”) using a series of sed commands. Renamed branches are found in the topology shown in Figure S9. Support values were then removed to create a simplified “wiped_tree” with clear taxonomic names. For each gene, a loop was used to generate pruned trees that included only the taxa relevant to that gene. The tree_doctor utility removed any taxa not present in the corresponding alignment file, ensuring that each pruned tree was gene-specific and consistent with the alignment. The pruned trees were saved for downstream analyses.

### Functional annotation and pathway enrichment analysis

To determine the function of genes of interest, we recovered GO (gene ontology) Terms using the UniProt Knowledgebase (UniProtKB; (UniProt Consortium 2021). For GO Term visualization we used Revigo. Revigo uses multidimensional scaling to reduce the dimensionality of a matrix of the GO terms pairwise semantic similarities. We used an enrichment analysis online tool (Enrichr) (Chen et al. 2013; Kuleshov et al. 2016) to compute gene set enrichment analysis against the libraries “KEGG human database” and “GO Biological Process 2023” to identify pathways and processes that were overrepresented among input genes. Enrichr uses a Fisher exact test, a developed z-score of the deviation from the expected rank by the Fisher exact test, and a combined score among *P*-values computed with the Fisher exact test with the z-score to assess the significance of the overlap between the input list and the gene sets present in the selected databases (Chen et al. 2013; Kuleshov et al. 2016).

### Demographic analysis

We reconstructed the demographic histories of the 12 chelonian species using the PSMC model (Li and Durbin 2011). Consensus sequences were generated from filtered gVCF files using BCFtools, and the resulting FASTA files were converted to the PSMC input format via fq2psmcfa. Initial assessments using default PSMC parameters (-N25 -t15 -r5 -p 4 + 25*2 + 4 + 6) revealed insufficient resolution for recent demographic events and potential overfitting of ancient intervals across several taxa (Figure S10). To address this, we re-analyzed the dataset using an optimized parameter set (-N25 -t10 -r5 -p 4*1 + 25*2 + 4 + 6). The modification of the time intervals (splitting the first parameter block) and reduction of time depth improved the signal-to-noise ratio and allowed for a standardized comparison of bottleneck timing across all samples. Results were scaled assuming a neutral mutation rate (μ) of 1.0 × 10^−8^ substitutions per site per generation. This value was selected as a representative intermediate between the rates previously estimated for *C. picta* (2.30 × 10^−10^) (Bergeron et al. 2023) and crocodilians (7.9 × 10^−9^) (Green et al. 2014). Species-specific generation times were derived from the midpoint of published estimates (Table S24).

### Evolutionary rates comparison

Evolutionary rates were estimated using our ultrametric species tree as a temporal reference. Divergence times (T) were calculated by determining the distance from the tips to their Most Recent Common Ancestor using the ape package in R (Paradis and Schliep 2019). A pairwise divergence age matrix was generated to provide the temporal denominator for rate calculations across all taxa. Substitution rates were calculated across 14,898 single-copy orthologous gene alignments (*Turtle dataset*). To account for the complexities of molecular evolution over deep time, two distinct distance models were employed: P-distance: A “raw” measure of the proportion of nucleotide sites at which two sequences differ. Kimura 2-Parameter (K2P): A more robust model that corrects for multiple substitutions at the same site and accounts for differing rates of transitions and transversions. For both models, the evolutionary rate (r) was calculated as: *r* = *D*/2*T*, where *D* represents the genetic distance and *T* represents the time since divergence in mya. Pairwise results were aggregated at the species level. Due to the inherent skewness of genomic rate data, where the majority of genes are highly conserved and a small subset evolve rapidly, all statistical analyses and visualizations were conducted using a log10 transformation. Final visualizations were produced using the K2P-derived rates. All analyses were implemented in R using the ape, tidyr (Wickham et al. 2014), dplyr (Wickham 2023) packages.

## Supplementary Material

msag114_Supplementary_Data

## Data Availability

Scripts and tutorials used for this study are available at https://github.com/ramoseks/Ramos_2026_MarineTurtleAdaptation. Assembly data IDs underlying this article are available in the online Supplementary material online.

## References

[msag114-B1] Agrawal AA . Toward a predictive framework for convergent evolution: integrating natural history, genetic mechanisms, and consequences for the diversity of life. Am Nat. 2017:190:S1–S12. 10.1086/692111. 28731831

[msag114-B2] Arantes LS et al Haplotype-resolved reference genomes of the sea turtle clade unveil ultra-syntenic genomes with hotspots of divergence. Gigascience. 2025:14:giaf105. 10.1093/gigascience/giaf105. 40971593 PMC12448945

[msag114-B3] Bartoli G et al Final closure of Panama and the onset of northern hemisphere glaciation. Earth Planet Sci Lett. 2005:237:33–44. 10.1016/j.epsl.2005.06.020.

[msag114-B4] Bentley BP et al Divergent sensory and immune gene evolution in sea turtles with contrasting demographic and life histories. Proc Natl Acad Sci U S A. 2023:120:e2201076120. 10.1073/pnas.2201076120. 36749728 PMC9962930

[msag114-B5] Bentley PJ, Bretz WL, Schmidt-Nielsen K. Osmoregulation in the diamondback terrapin, *Malaclemys terrapin centrata*. J Exp Biol. 1967:46:161–167. 10.1242/jeb.46.1.161. 6032170

[msag114-B6] Bergeron LA et al Evolution of the germline mutation rate across vertebrates. Nature. 2023:615:285–291. 10.1038/s41586-023-05752-y. 36859541 PMC9995274

[msag114-B7] Bigham AW et al Identifying positive selection candidate loci for high-altitude adaptation in Andean populations. Hum Genomics. 2009:4:79–90. 10.1186/1479-7364-4-2-79. 20038496 PMC2857381

[msag114-B8] Blumer M et al Gene losses in the common vampire bat illuminate molecular adaptations to blood feeding. Sci Adv. 2022:8:eabm6494. 10.1126/sciadv.abm6494. 35333583 PMC8956264

[msag114-B9] Brian Simison W, Parham JF, Papenfuss TJ, Lam AW, Henderson JB. An annotated chromosome-level reference genome of the red-eared slider turtle (*Trachemys scripta elegans*). Genome Biol Evol. 2020:12:456–462. 10.1093/gbe/evaa063. 32227195 PMC7186784

[msag114-B10] Bridgham JT . Predicting the basis of convergent evolution. Science. 2016:354:289. 10.1126/science.aai7394. 27846519

[msag114-B11] Brownstein CD et al The genomic signatures of evolutionary stasis. Evolution. 2024:78:821–834. 10.1093/evolut/qpae028. 38437861

[msag114-B12] Cao D, Wang M, Ge Y, Gong S. Draft genome of the big-headed turtle *Platysternon megacephalum*. Sci Data. 2019:6:60. 10.1038/s41597-019-0067-9. 31097710 PMC6522511

[msag114-B13] Capella-Gutiérrez S, Silla-Martínez JM, Gabaldón T. Trimal: a tool for automated alignment trimming in large-scale phylogenetic analyses. Bioinformatics. 2009:25:1972–1973. 10.1093/bioinformatics/btp348. 19505945 PMC2712344

[msag114-B14] Castellini MA, Mellish J-A. Marine mammal physiology: requisites for ocean living. CRC Press; 2015.

[msag114-B15] Chen EY et al Enrichr: interactive and collaborative HTML5 gene list enrichment analysis tool. BMC Bioinformatics. 2013:14:128. 10.1186/1471-2105-14-128. 23586463 PMC3637064

[msag114-B16] Chiari Y, Cahais V, Galtier N, Delsuc F. Phylogenomic analyses support the position of turtles as the sister group of birds and crocodiles (Archosauria). BMC Biol. 2012:10:65. 10.1186/1741-7007-10-65. 22839781 PMC3473239

[msag114-B17] Chikina M, Robinson JD, Clark NL. Hundreds of genes experienced convergent shifts in selective pressure in marine mammals. Mol Biol Evol. 2016:33:2182–2192. 10.1093/molbev/msw112. 27329977 PMC5854031

[msag114-B18] Chow JC, Anderson PE, Shedlock AM. Sea turtle population genomic discovery: global and locus-specific signatures of polymorphism, selection, and adaptive potential. Genome Biol Evol. 2019:11:2797–2806. 10.1093/gbe/evz190. 31504487 PMC6786478

[msag114-B19] Colautti RI, Lau JA. Contemporary evolution during invasion: evidence for differentiation, natural selection, and local adaptation. Mol Ecol. 2015:24:1999–2017. 10.1111/mec.13162. 25891044

[msag114-B20] Cole TL et al Genomic insights into the secondary aquatic transition of penguins. Nat Commun. 2022:13:3912. 10.1038/s41467-022-31508-9. 35853876 PMC9296559

[msag114-B21] Cramp RL, Meyer EA, Sparks N, Franklin CE. Functional and morphological plasticity of crocodile (*Crocodylus porosus*) salt glands. J Exp Biol. 2008:211:1482–1489. 10.1242/jeb.015636. 18424682

[msag114-B22] Crawford NG et al A phylogenomic analysis of turtles. Mol Phylogenet Evol. 2015:83:250–257. 10.1016/j.ympev.2014.10.021. 25450099

[msag114-B23] Dasmeh P, Serohijos AWR, Kepp KP, Shakhnovich EI. Positively selected sites in cetacean myoglobins contribute to protein stability. PLoS Comput Biol. 2013:9:e1002929. 10.1371/journal.pcbi.1002929. 23505347 PMC3591298

[msag114-B24] Davies KTJ, Bennett NC, Tsagkogeorga G, Rossiter SJ, Faulkes CG. Family wide molecular adaptations to underground life in African mole-rats revealed by phylogenomic analysis. Mol Biol Evol. 2015:32:3089–3107. 10.1093/molbev/msv175. 26318402 PMC4652621

[msag114-B25] Di Franco A, Poujol R, Baurain D, Philippe H. Evaluating the usefulness of alignment filtering methods to reduce the impact of errors on evolutionary inferences. BMC Evol Biol. 2019:19:21. 10.1186/s12862-019-1350-2. 30634908 PMC6330419

[msag114-B26] Dong K et al Genomic scan reveals loci under altitude adaptation in Tibetan and Dahe pigs. PLoS One. 2014:9:e110520. 10.1371/journal.pone.0110520. 25329542 PMC4201535

[msag114-B27] Duchene S et al Marine turtle mitogenome phylogenetics and evolution. Mol Phylogenet Evol. 2012:65:241–250. 10.1016/j.ympev.2012.06.010. 22750111

[msag114-B28] Ellegren H . Comparative genomics and the study of evolution by natural selection. Mol Ecol. 2008:17:4586–4596. 10.1111/j.1365-294X.2008.03954.x. 19140982

[msag114-B29] Emms DM, Kelly S. OrthoFinder: solving fundamental biases in whole genome comparisons dramatically improves orthogroup inference accuracy. Genome Biol. 2015:16:157. 10.1186/s13059-015-0721-2. 26243257 PMC4531804

[msag114-B30] Emms DM, Kelly S. OrthoFinder: phylogenetic orthology inference for comparative genomics. Genome Biol. 2019:20:238. 10.1186/s13059-019-1832-y. 31727128 PMC6857279

[msag114-B31] Enard D, Messer PW, Petrov DA. Genome-wide signals of positive selection in human evolution. Genome Res. 2014:24:885–895. 10.1101/gr.164822.113. 24619126 PMC4032853

[msag114-B32] Escalona T, Weadick CJ, Antunes A. Adaptive patterns of mitogenome evolution are associated with the loss of shell Scutes in turtles. Mol Biol Evol. 2017:34:2522–2536. 10.1093/molbev/msx167. 28591857 PMC6298445

[msag114-B33] Evers SW, Benson RBJ. A new phylogenetic hypothesis of turtles with implications for the timing and number of evolutionary transitions to marine lifestyles in the group. Palaeontology. 2019:62:93–134. 10.1111/pala.12384.

[msag114-B34] Flynn JM et al RepeatModeler2: automated genomic discovery of transposable element families. Proc Natl Acad Sci U S A. 2020:117:9451–9457. 10.1073/pnas.1921046117. 32300014 PMC7196820

[msag114-B35] Foley NM et al Growing old, yet staying young: the role of telomeres in bats’ exceptional longevity. Sci Adv. 2018:4:eaao0926. 10.1126/sciadv.aao0926. 29441358 PMC5810611

[msag114-B36] Foote AD et al Convergent evolution of the genomes of marine mammals. Nat Genet. 2015:47:272–275. 10.1038/ng.3198. 25621460 PMC4644735

[msag114-B37] Fossette S et al Behaviour and buoyancy regulation in the deepest-diving reptile: the leatherback turtle. J Exp Biol. 2010:213:4074–4083. 10.1242/jeb.048207. 21075949

[msag114-B38] Ghosh A et al A high-quality reference genome assembly of the saltwater crocodile, Crocodylus porosus, reveals patterns of selection in Crocodylidae. Genome Biol Evol. 2020:12:3635–3646. 10.1093/gbe/evz269. 31821505 PMC6946029

[msag114-B39] Glaberman S, Bulls SE, Vazquez JM, Chiari Y, Lynch VJ. Concurrent evolution of antiaging gene duplications and cellular phenotypes in long-lived turtles. Genome Biol Evol. 2021:13:evab244. 10.1093/gbe/evab244. 34792580 PMC8688777

[msag114-B40] Green RE et al Three crocodilian genomes reveal ancestral patterns of evolution among archosaurs. Science. 2014:346:1254449. 10.1126/science.1254449. 25504731 PMC4386873

[msag114-B41] Guan D et al Identifying and removing haplotypic duplication in primary genome assemblies. Bioinformatics. 2020:36:2896–2898. 10.1093/bioinformatics/btaa025. 31971576 PMC7203741

[msag114-B42] Guang-Xin E et al Genome-wide selective sweep analysis of the high-altitude adaptability of yaks by using the copy number variant. 3 Biotech. 2020:10:259. 10.1007/s13205-020-02254-w. PMC7235113 32432020

[msag114-B43] Guillon J-M, Guéry L, Hulin V, Girondot M. A large phylogeny of turtles (Testudines) using molecular data. Contrib. Zool. 2012:81:147–158j. 10.1163/18759866-08103002.

[msag114-B44] Harada Y, Fujisawa Y, Takata R, Shuin T, Miki T, Fujioka T, Nakamura Y, Katagiri T. Cell-permeable peptide DEPDC1-ZNF224 interferes with transcriptional repression and oncogenicity in bladder cancer cells. Cancer Res. 2010:15:5829–5839. 10.1158/0008-5472.CAN-10-0255. 20587513

[msag114-B45] Herrera-Álvarez S, Karlsson E, Ryder OA, Lindblad-Toh K, Crawford AJ. How to make a rodent giant: genomic basis and tradeoffs of gigantism in the capybara, the World's largest rodent. Mol Biol Evol. 2021:38:1715–1730. 10.1093/molbev/msaa285. 33169792 PMC8097284

[msag114-B46] Hilgers L et al Avoidable false PSMC population size peaks occur across numerous studies. Curr Biol. 2025:35:927–930.e3. 10.1016/j.cub.2024.09.028. 39919744

[msag114-B47] Hill J et al Recurrent convergent evolution at amino acid residue 261 in fish rhodopsin. Proc Natl Acad Sci U S A. 2019:116:18473–18478. 10.1073/pnas.1908332116. 31451650 PMC6744887

[msag114-B48] Houssaye A, Fish FE. Functional (secondary) adaptation to an aquatic life in vertebrates: an introduction to the symposium. Integr Comp Biol. 2016:56:1266–1270. 10.1093/icb/icw129. 27940617

[msag114-B49] Hu Y et al Comparative genomics reveals convergent evolution between the bamboo-eating giant and red pandas. Proc Natl Acad Sci U S A. 2017:114:1081–1086. 10.1073/pnas.1613870114. 28096377 PMC5293045

[msag114-B50] Hubisz MJ, Pollard KS, Siepel A. PHAST and RPHAST: phylogenetic analysis with space/time models. Brief Bioinform. 2011:12:41–51. 10.1093/bib/bbq072. 21278375 PMC3030812

[msag114-B51] Huelsmann M et al Genes lost during the transition from land to water in cetaceans highlight genomic changes associated with aquatic adaptations. Sci Adv. 2019:5:eaaw6671. 10.1126/sciadv.aaw6671. 31579821 PMC6760925

[msag114-B52] Hughes AL . Looking for Darwin in all the wrong places: the misguided quest for positive selection at the nucleotide sequence level. Heredity (Edinb). 2007:99:364–373. 10.1038/sj.hdy.6801031. 17622265

[msag114-B53] Irisarri I et al Phylotranscriptomic consolidation of the jawed vertebrate timetree. Nat Ecol Evol. 2017:1:1370–1378. 10.1038/s41559-017-0240-5. 28890940 PMC5584656

[msag114-B54] Jones K, Ariel E, Burgess G, Read M. A review of fibropapillomatosis in green turtles (*Chelonia mydas*). Vet. J. 2016:212:48–57. 10.1016/j.tvjl.2015.10.041. 27256025

[msag114-B55] Joyce WG, Parham JF, Lyson TR, Warnock RCM, Donoghue PCJ. A divergence dating analysis of turtles using fossil calibrations: an example of best practices. J. Paleontol. 2013:87:612–634. 10.1666/12-149.

[msag114-B56] Junker N, Gossmann TI. Adaptation-driven evolution of Sirtuin 1 (SIRT1), a key regulator of metabolism and aging, in marmot Species. Front Ecol Evol. 2021:9:666564. 10.3389/fevo.2021.666564.

[msag114-B57] Kaplinsky NJ et al The embryonic transcriptome of the red-eared slider turtle (Trachemys scripta). PLoS One. 2013:8:e66357. 10.1371/journal.pone.0066357. 23840449 PMC3686863

[msag114-B58] Katoh K, Standley DM. MAFFT multiple sequence alignment software version 7: improvements in performance and usability. Mol Biol Evol. 2013:30:772–780. 10.1093/molbev/mst010. 23329690 PMC3603318

[msag114-B59] Keane M et al Insights into the evolution of longevity from the bowhead whale genome. Cell Rep. 2015:10:112–122. 10.1016/j.celrep.2014.12.008. 25565328 PMC4536333

[msag114-B60] Kelley JL, Brown AP, Therkildsen NO, Foote AD. The life aquatic: advances in marine vertebrate genomics. Nat Rev Genet. 2016:17:523–534. 10.1038/nrg.2016.66. 27376488

[msag114-B61] Kim EB et al Genome sequencing reveals insights into physiology and longevity of the naked mole rat. Nature. 2011:479:223–227. 10.1038/nature10533. 21993625 PMC3319411

[msag114-B62] Kim Y, Stephan W. Detecting a local signature of genetic Hitchhiking along a recombining chromosome. Genetics. 2002:160:765–777. 10.1093/genetics/160.2.765. 11861577 PMC1461968

[msag114-B63] Kirilenko BM et al Integrating gene annotation with orthology inference at scale. Science. 2023:380:eabn3107. 10.1126/science.abn3107. 37104600 PMC10193443

[msag114-B64] Kishida T et al Loss of olfaction in sea snakes provides new perspectives on the aquatic adaptation of amniotes. Proc Biol Sci. 2019:286:20191828. 10.1098/rspb.2019.1828. 31506057 PMC6742997

[msag114-B65] Kishida T, Thewissen J, Hayakawa T, Imai H, Agata K. Aquatic adaptation and the evolution of smell and taste in whales. Zoological Lett. 2015:1:9. 10.1186/s40851-014-0002-z. 26605054 PMC4604112

[msag114-B66] Kosakovsky Pond SL, et al HyPhy 2.5—a customizable platform for evolutionary hypothesis testing using phylogenies. Molecular Biology and Evolution. 2020:37:295–299. 10.1093/molbev/msz197. 31504749 PMC8204705

[msag114-B67] Kuleshov MV et al Enrichr: a comprehensive gene set enrichment analysis web server 2016 update. Nucleic Acids Res. 2016:44:W90–W97. 10.1093/nar/gkw377. 27141961 PMC4987924

[msag114-B68] Lee A, Beck L, Markovich D. The human renal sodium sulfate cotransporter (SLC13A1; hNaSi-1) cDNA and gene: organization, chromosomal localization, and functional characterization. Genomics. 2000:70:354–363. 10.1006/geno.2000.6404. 11161786

[msag114-B69] León F et al Comparative genomics supports ecologically induced selection as a putative driver of banded penguin diversification. Mol Biol Evol. 2024:41:msae166. 10.1093/molbev/msae166. 39150953 PMC11371425

[msag114-B70] Li C, Wu X-C, Rieppel O, Wang L-T, Zhao L-J. An ancestral turtle from the late Triassic of southwestern China. Nature. 2008:456:497–501. 10.1038/nature07533. 19037315

[msag114-B71] Li H, Durbin R. Inference of human population history from individual whole-genome sequences. Nature. 2011:475:493–496. 10.1038/nature10231. 21753753 PMC3154645

[msag114-B72] Li Z et al RNA-Seq improves annotation of protein-coding genes in the cucumber genome. BMC Genomics. 2011:12:540. 10.1186/1471-2164-12-540. 22047402 PMC3219749

[msag114-B73] Liu A et al Convergent degeneration of olfactory receptor gene repertoires in marine mammals. BMC Genomics. 2019:20:977. 10.1186/s12864-019-6290-0. 31842731 PMC6916060

[msag114-B74] Liu J et al Genetic signatures of high-altitude adaptation and geographic distribution in Tibetan sheep. Sci Rep. 2020:10:18332. 10.1038/s41598-020-75428-4. 33110149 PMC7591910

[msag114-B75] Liu J et al Chromosome-level genome assembly of the Chinese three-keeled pond turtle (*Mauremys reevesii*) provides insights into freshwater adaptation. Mol Ecol Resour. 2022:22:1596–1605. 10.1111/1755-0998.13563. 34845835

[msag114-B76] Lovich JE, Gibbons W. Turtles of the world: a guide to every family. 2021. https://www.torrossa.com/gs/resourceProxy?an=5623097&publisher=FZO137

[msag114-B77] Ludington AJ, Hammond JM, Breen J, Deveson IW, Sanders KL. New chromosome-scale genomes provide insights into marine adaptations of sea snakes (Hydrophis: Elapidae). BMC Biol. 2023:21:284. 10.1186/s12915-023-01772-2. 38066641 PMC10709897

[msag114-B78] Lunt J, Smee DL. Turbidity alters estuarine biodiversity and species composition. ICES J Mar Sci. 2020:77:379–387. 10.1093/icesjms/fsz214.

[msag114-B79] Lutcavage ME, Bushnell PG, Jones DR. Oxygen stores and aerobic metabolism in the leatherback sea turtle. Can J Zool. 1992:70:348–351. 10.1139/z92-051.

[msag114-B80] Lutz P . Salt, water, and pH balance in the sea turtle. In: Musick JA, Boca Raton FL, editors. The Biology of Sea Turtles. Vol. 1. CRC Press: 2017. p. 343–361.

[msag114-B81] Lutz PL, Lutcavage ME. Diving physiology. In: Musick JA, Boca Raton FL, editors. The biology of sea turtles. Vol. 1. CRC Press: 2017. p. 291–310.

[msag114-B82] Martin SH, Van Belleghem SM. Exploring evolutionary relationships across the genome using topology weighting. Genetics. 2017:206:429–438. 10.1534/genetics.116.194720. 28341652 PMC5419486

[msag114-B83] Mazet O, Rodríguez W, Grusea S, Boitard S, Chikhi L. On the importance of being structured: instantaneous coalescence rates and human evolution–lessons for ancestral population size inference? Heredity (Edinb). 2016:116:362–371. 10.1038/hdy.2015.104. 26647653 PMC4806692

[msag114-B84] McGowen MR, Grossman LI, Wildman DE. Dolphin genome provides evidence for adaptive evolution of nervous system genes and a molecular rate slowdown. Proc Biol Sci. 2012:279:3643–3651. 10.1098/rspb.2012.0869. 22740643 PMC3415902

[msag114-B85] Mendes FK, Vanderpool D, Fulton B, Hahn MW. CAFE 5 models variation in evolutionary rates among gene families. Bioinformatics. 2020:36:5516–5518. 10.1093/bioinformatics/btaa1022. 33325502

[msag114-B86] Minh BQ et al IQ-TREE 2: new models and efficient methods for phylogenetic inference in the genomic era. Mol Biol Evol. 2020:37:1530–1534. 10.1093/molbev/msaa015. 32011700 PMC7182206

[msag114-B87] Montgomery SH, Mundy NI, Barton RA. ASPM and mammalian brain evolution: a case study in the difficulty in making macroevolutionary inferences about gene–phenotype associations. Proc R Soc Lond B Biol Sci. 2014:281:20131743. 10.1098/rspb.2013.1743. PMC3906929 24452019

[msag114-B88] Morales AE et al Distinct genes with similar functions underlie convergent evolution in Myotis bat ecomorphs. Mol Biol Evol. 2024:41:msae165. 10.1093/molbev/msae165. 39116340 PMC11371419

[msag114-B89] Moreno JM, Jesus TF, Coelho MM, Sousa VC. Adaptation and convergence in circadian-related genes in Iberian freshwater fish. BMC Ecol Evol. 2021:21:38. 10.1186/s12862-021-01767-z. 33685402 PMC7941933

[msag114-B90] Moreno Santillán DD et al Large-scale genome sampling reveals unique immunity and metabolic adaptations in bats. Mol Ecol. 2021:30:6449–6467. 10.1111/mec.16027. 34146369

[msag114-B91] Motani R, Vermeij GJ. Ecophysiological steps of marine adaptation in extant and extinct non-avian tetrapods. Biol Rev Camb Philos Soc. 2021:96:1769–1798. 10.1111/brv.12724. 33904243

[msag114-B92] Murrell B et al Gene-wide identification of episodic selection. Mol Biol Evol. 2015:32:1365–1371. 10.1093/molbev/msv035. 25701167 PMC4408417

[msag114-B93] Nery MF, Arroyo JI, Opazo JC. Increased rate of hair keratin gene loss in the cetacean lineage. BMC Genomics. 2014:15:869. 10.1186/1471-2164-15-869. 25287022 PMC4195889

[msag114-B94] Newell RC . Adaptation to environment: essays on the physiology of marine animals. Elsevier; 2013.

[msag114-B95] Ng CKY et al Overview of the population genetics and connectivity of sea turtles in the east Asia region and their conservation implications. Front Mar Sci. 2024:11:1325849. 10.3389/fmars.2024.1325849.

[msag114-B96] Ortiz RM . Osmoregulation in marine mammals. J Exp Biol. 2001:204:1831–1844. 10.1242/jeb.204.11.1831. 11441026

[msag114-B97] Pacini N, Harper DM. 6—Aquatic, Semi-aquatic and riparian vertebrates. In: Dudgeon D, editor. Tropical stream ecology. Academic Press; 2008. p. 147–197.

[msag114-B98] Page-Karjian A . Fibropapillomatosis in marine turtles. In: Miller RE, Lamberski N, Calle. PP, editors. Fowler's zoo and wild animal medicine current therapy. Vol. 9. Elsevier: 2019. p. 398–403.

[msag114-B99] Paladino FV, O’Connor MP, Spotila JR. Metabolism of leatherback turtles, gigantothermy, and thermoregulation of dinosaurs. Nature. 1990:344:858–860. 10.1038/344858a0.

[msag114-B100] Paradis E, Schliep K. Ape 5.0: an environment for modern phylogenetics and evolutionary analyses in R. Bioinformatics. 2019:35:526–528. 10.1093/bioinformatics/bty633. 30016406

[msag114-B101] Peng C et al The genome of Shaw's sea snake (Hydrophis curtus) reveals secondary adaptation to its marine environment. Mol Biol Evol. 2020:37:1744–1760. 10.1093/molbev/msaa043. 32077944

[msag114-B102] Pertea G, Pertea M. GFF utilities: GffRead and GffCompare. F1000Res. 2020:9:e304. 10.12688/f1000research.23297.2. PMC7222033 32489650

[msag114-B103] Piotrowski ER et al Ontogeny of carbon monoxide-related gene expression in a deep-diving marine mammal. Front Physiol. 2021:12:762102. 10.3389/fphys.2021.762102. 34744798 PMC8567018

[msag114-B104] Policarpo M, Baldwin MW, Casane D, Salzburger W. Diversity and evolution of the vertebrate chemoreceptor gene repertoire. Nat Commun. 2024:15:1421. 10.1038/s41467-024-45500-y. 38360851 PMC10869828

[msag114-B105] Pond SLK, Frost SDW, Muse SV. Hyphy: hypothesis testing using phylogenies. Bioinformatics. 2005:21:676–679. 10.1093/bioinformatics/bti079. 15509596

[msag114-B106] Pyenson ND, Kelley NP, Parham JF. Marine tetrapod macroevolution: physical and biological drivers on 250 Ma of invasions and evolution in ocean ecosystems. Palaeogeogr Palaeoclimatol Palaeoecol. 2014:400:1–8. 10.1016/j.palaeo.2014.02.018.

[msag114-B107] Quesada V et al Giant tortoise genomes provide insights into longevity and age-related disease. Nat Ecol Evol. 2019:3:87–95. 10.1038/s41559-018-0733-x. 30510174 PMC6314442

[msag114-B108] Ramos EKS, Freitas L, Nery MF. The role of selection in the evolution of marine turtles mitogenomes. Sci Rep. 2020:10:1–13. 10.1038/s41598-019-56847-4. 33046778 PMC7550602

[msag114-B109] Ranallo-Benavidez TR, Jaron KS, Schatz MC. GenomeScope 2.0 and Smudgeplot for reference-free profiling of polyploid genomes. Nat Commun. 2020:11:1432. 10.1038/s41467-020-14998-3. 32188846 PMC7080791

[msag114-B110] Ranwez V, Douzery EJP, Cambon C, Chantret N, Delsuc F. MACSE v2: toolkit for the alignment of coding sequences accounting for frameshifts and stop codons. Mol Biol Evol. 2018:35:2582–2584. 10.1093/molbev/msy159. 30165589 PMC6188553

[msag114-B111] R Core Team . R: a language and environment for statistical computing. R Foundation for 1069 Statistical Computing; 2023; 2023. [updated 2005 May-Jul]. https://www.r-project.org/.

[msag114-B112] Reznick DN, Ghalambor CK. The population ecology of contemporary adaptations: what empirical studies reveal about the conditions that promote adaptive evolution. Genetica. 2001:112-113:183–198. 10.1023/A:1013352109042. 11838765

[msag114-B113] Rhie A et al Towards complete and error-free genome assemblies of all vertebrate species. Nature. 2021:592:737–746. 10.1038/s41586-021-03451-0. 33911273 PMC8081667

[msag114-B114] Rhie A, Walenz BP, Koren S, Phillippy AM. Merqury: reference-free quality, completeness, and phasing assessment for genome assemblies. Genome Biol. 2020:21:245. 10.1186/s13059-020-02134-9. 32928274 PMC7488777

[msag114-B115] Rodríguez-Zárate CJ, Rocha-Olivares A, Beheregaray LB. Genetic signature of a recent metapopulation bottleneck in the olive ridley turtle (Lepidochelys olivacea) after intensive commercial exploitation in Mexico. Biol Conserv. 2013:168:10–18. 10.1016/j.biocon.2013.09.009.

[msag114-B116] Saito S et al Characterization of TRPA channels in the starfish Patiria pectinifera: involvement of thermally activated TRPA1 in thermotaxis in marine planktonic larvae. Sci Rep. 2017:7:2173. 10.1038/s41598-017-02171-8. 28526851 PMC5438368

[msag114-B117] Sakaguchi M, Huh N-H. S100a11, a dual growth regulator of epidermal keratinocytes. Amino Acids. 2011:41:797–807. 10.1007/s00726-010-0747-4. 20872027

[msag114-B118] Sanderson MJ . R8s: inferring absolute rates of molecular evolution and divergence times in the absence of a molecular clock. Bioinformatics. 2003:19:301–302. 10.1093/bioinformatics/19.2.301. 12538260

[msag114-B119] Sarangi GK, Romagné F, Castellano S. Distinct patterns of selection in selenium-dependent genes between land and aquatic vertebrates. Mol Biol Evol. 2018:35:1744–1756. 10.1093/molbev/msy070. 29669130

[msag114-B120] Seiko T et al Visual adaptation of opsin genes to the aquatic environment in sea snakes. BMC Evol Biol. 2020:20:158. 10.1186/s12862-020-01725-1. 33243140 PMC7690139

[msag114-B121] Shaffer HB et al The western painted turtle genome, a model for the evolution of extreme physiological adaptations in a slowly evolving lineage. Genome Biol. 2013:14:R28. 10.1186/gb-2013-14-3-r28. 23537068 PMC4054807

[msag114-B122] Shaffer HB, McCartney-Melstad E, Near TJ, Mount GG, Spinks PQ. Phylogenomic analyses of 539 highly informative loci dates a fully resolved time tree for the major clades of living turtles (testudines). Mol Phylogenet Evol. 2017:115:7–15. 10.1016/j.ympev.2017.07.006. 28711671

[msag114-B123] Simão FA, Waterhouse RM, Ioannidis P, Kriventseva EV, Zdobnov EM. BUSCO: assessing genome assembly and annotation completeness with single-copy orthologs. Bioinformatics. 2015:31:3210–3212. 10.1093/bioinformatics/btv351. 26059717

[msag114-B124] Simões BF et al Spectral diversification and trans-species Allelic polymorphism during the land-to-sea transition in snakes. Curr Biol. 2020:30:2608–2615.e4. 10.1016/j.cub.2020.04.061. 32470360

[msag114-B125] Smith MD et al Less is more: an adaptive branch-site random effects model for efficient detection of episodic diversifying selection. Mol Biol Evol. 2015:32:1342–1353. 10.1093/molbev/msv022. 25697341 PMC4408413

[msag114-B126] Southwood AL et al Heart rates and diving behavior of leatherback sea turtles in the eastern Pacific Ocean. J Exp Biol. 1999:202:1115–1125. 10.1242/jeb.202.9.1115. 10101109

[msag114-B127] Southwood Williard A, Harden LA, Jones TT, Midway SR. Effects of temperature and salinity on body fluid dynamics and metabolism in the estuarine diamondback terrapin (*Malaclemys terrapin*). J Exp Biol. 2019:222:jeb202390. 10.1242/jeb.202390. 31064853

[msag114-B128] Spielman SJ et al Evolution of viral genomes: interplay between selection, recombination, and other forces. Methods Mol. Biol. 2019:1910:427–468. 10.1007/978-1-4939-9074-0_14. 31278673

[msag114-B129] Storz JF . Causes of molecular convergence and parallelism in protein evolution. Nat Rev Genet. 2016:17:239–250. 10.1038/nrg.2016.11. 26972590 PMC5482790

[msag114-B130] Stroud JT, Losos JB. Ecological opportunity and adaptive radiation. Annu Rev Ecol Evol Syst. 2016:47:507–532. 10.1146/annurev-ecolsys-121415-032254.

[msag114-B131] Sullivan J, De Panis D, Galeone V, Mazzoni CJ. Genome evaluation pipeline (GEP): a fully-automated quality control tool for parallel evaluation of genome assemblies. Bioinform. Adv. 2025:5:vbaf147. 10.1093/bioadv/vbaf147. PMC12296351 40718017

[msag114-B132] Sun X et al Comparative genomics analyses of alpha-keratins reveal insights into evolutionary adaptation of marine mammals. Front Zool. 2017:14:41. 10.1186/s12983-017-0225-x. 28785294 PMC5540548

[msag114-B133] Sun Y-B et al Genome-wide scans for candidate genes involved in the aquatic adaptation of dolphins. Genome Biol Evol. 2013:5:130–139. 10.1093/gbe/evs123. 23246795 PMC3595024

[msag114-B134] Tajima F . The effect of change in population size on DNA polymorphism. Genetics. 1989:123:597–601. 10.1093/genetics/123.3.597. 2599369 PMC1203832

[msag114-B135] Tarailo-Graovac M, Chen N. Using RepeatMasker to identify repetitive elements in genomic sequences. Curr Protoc Bioinformatics. 2009. Chapter4:Unit 4.10. 10.1002/0471250953.bi0410s25. 19274634

[msag114-B136] Thibaud-Nissen F, Souvorov A, Murphy TD, DiCuccio M, Kitts P. Eukaryotic genome annotation pipeline. Vol. 2. The NCBI handbook; 2013. https://www.ncbi.nlm.nih.gov/sites/books/NBK169439/pdf/Bookshelf_NBK169439.pdf.

[msag114-B137] Thomson RC, Spinks PQ, Shaffer HB. A global phylogeny of turtles reveals a burst of climate-associated diversification on continental margins. Proc Natl Acad Sci U S A. 2021:118:e2012215118. 10.1073/pnas.2012215118. 33558231 PMC7896334

[msag114-B138] Tian R et al Molecular evolution of vision-related genes may contribute to marsupial photic niche adaptations. Front Ecol Evol. 2022:10:982073. 10.3389/fevo.2022.982073.

[msag114-B139] Tollis M et al The Agassiz's desert tortoise genome provides a resource for the conservation of a threatened species. PLoS One. 2017:12:e0177708. 10.1371/journal.pone.0177708. 28562605 PMC5451010

[msag114-B140] Turtle Taxonomy Working Group (TTWG) et al Turtles of the world: annotated checklist and atlas of taxonomy, synonymy, distribution, and conservation status. In: Rhodin AGJ, Iverson JB, van Dijk PP, Stanford CB, Goode EV, Buhlmann KA, Mittermeier RA, editors. Conservation biology of freshwater turtles and tortoises: a compilation project of the IUCN SSC tortoise and freshwater turtle specialist group. 10th ed. Chelonian Research Monographs; 2025. p. 1–575.

[msag114-B141] UniProt Consortium . UniProt: the universal protein knowledgebase in 2021. Nucleic Acids Res. 2021:49:D480–D489. 10.1093/nar/gkaa1100. 33237286 PMC7778908

[msag114-B142] Vermeij GJ, Motani R. Land to sea transitions in vertebrates: the dynamics of colonization. Paleobiology. 2018:44:237–250. 10.1017/pab.2017.37.

[msag114-B143] Vianna JA et al Genome-wide analyses reveal drivers of penguin diversification. Proc Natl Acad Sci U S A. 2020:117:22303–22310. 10.1073/pnas.2006659117. 32817535 PMC7486704

[msag114-B144] Vuruputoor VS et al Welcome to the big leaves: best practices for improving genome annotation in non-model plant genomes. Appl Plant Sci. 2023:11:e11533. 10.1002/aps3.11533. 37601314 PMC10439824

[msag114-B145] Wang Z et al The draft genomes of soft-shell turtle and green sea turtle yield insights into the development and evolution of the turtle-specific body plan. Nat Genet. 2013:45:701–706. 10.1038/ng.2615. 23624526 PMC4000948

[msag114-B146] Welch AJ et al Polar bears exhibit genome-wide signatures of bioenergetic adaptation to life in the Arctic environment. Genome Biol Evol. 2014:6:433–450. 10.1093/gbe/evu025. 24504087 PMC3942037

[msag114-B147] Wickham H . dplyr: A grammar of data manipulation. 2023. https://user2014.r-project.org/abstracts/talks/45_Wickham.pdf.

[msag114-B148] Wickham H . Tidy Data. Journal of Statistical Software. 2014:59:1–23. 10.18637/jss.v059.i10.26917999

[msag114-B149] Xin J et al Chromatin accessibility landscape and regulatory network of high-altitude hypoxia adaptation. Nat Commun. 2020:11:4928. 10.1038/s41467-020-18638-8. 33004791 PMC7529806

[msag114-B150] Xu S et al Positive selection at the ASPM gene coincides with brain size enlargements in cetaceans. Proc Biol Sci. 2012:279:4433–4440. 10.1098/rspb.2012.1729. 22977148 PMC3479811

[msag114-B151] Yang B, Song Y, Zhao D, Verkman AS. Phenotype analysis of aquaporin-8 null mice. Am J Physiol Cell Physiol. 2005a:288:C1161–C1170. 10.1152/ajpcell.00564.2004. 15647389

[msag114-B152] Yang Z, Wong WSW, Nielsen R. Bayes empirical Bayes inference of amino acid sites under positive selection. Mol Biol Evol. 2005b:22:1107–1118. 10.1093/molbev/msi097. 15689528

[msag114-B153] Ye J et al TRF2 and apollo cooperate with topoisomerase 2alpha to protect human telomeres from replicative damage. Cell. 2010:142:230–242. 10.1016/j.cell.2010.05.032. 20655466

[msag114-B154] Yim H-S et al Minke whale genome and aquatic adaptation in cetaceans. Nat Genet. 2014:46:88–92. 10.1038/ng.2835. 24270359 PMC4079537

[msag114-B155] Yuan Y et al Comparative genomics provides insights into the aquatic adaptations of mammals. Proc Natl Acad Sci U S A. 2021:118:e2106080118. 10.1073/pnas.2106080118. 34503999 PMC8449357

[msag114-B156] Zardoya R, Meyer A. The evolutionary position of turtles revised. Naturwissenschaften. 2001:88:193–200. 10.1007/s001140100228. 11482432

[msag114-B157] Zhang H et al Adaptive evolution of low-salinity tolerance and hypoosmotic regulation in a euryhaline teleost, takifugu obscurus. Mar Biol. 2020:167:90. 10.1007/s00227-020-03705-x.

[msag114-B158] Zhang J, Nielsen R, Yang Z. Evaluation of an improved branch-site likelihood method for detecting positive selection at the molecular level. Mol Biol Evol. 2005:22:2472–2479. 10.1093/molbev/msi237. 16107592

[msag114-B159] Zheng D et al PSTK is a novel gene associated with early lung injury in Paraquat Poisoning. Life Sci. 2015:123:9–17. 10.1016/j.lfs.2014.12.023. 25592138

[msag114-B160] Zhou X et al Molecular footprints of aquatic adaptation including bone mass changes in cetaceans. Genome Biol Evol. 2018:10:967–975. 10.1093/gbe/evy062. 29608729 PMC5952927

[msag114-B161] Zhou X, Seim I, Gladyshev VN. Convergent evolution of marine mammals is associated with distinct substitutions in common genes. Sci Rep. 2015:5:16550. 10.1038/srep16550. 26549748 PMC4637874

[msag114-B162] Zhu K et al The loss of taste genes in cetaceans. BMC Evol Biol. 2014:14:218. 10.1186/s12862-014-0218-8. 25305673 PMC4232718

