# Supplementary material for: The genomic basis of independent marine transitions in turtles: convergent episodic adaptation and demographic shifts
Source: Mol Biol Evol. 2026 May 6;43(5):msag114. doi: 10.1093/molbev/msag114 (PMC13221643; doi:10.1093/molbev/msag114)
Supplement: msag114_Supplementary_Data [file msag114_supplementary_data.zip › v6_Ramos_et_al_2026_Supplementary_Figures.pdf]

Supplementary Figures

The Genomic Basis of Independent Marine Transitions in Turtles: Convergent Episodic Adaptation and Demographic Shifts

Elisa Ramos[1,2], Leon Hilgers [3,4], Tomás Carrasco-Valenzuela[1,5], Diego De Panis[1], Agnello Picorelli[2], James Sullivan[1], Marcela Uliano-Silva[6,7], Blair P. Bentley[8], Peter A. Scott[9], Michael Hiller[3], Mariana F. Nery[2], H. Bradley Shaffer[10,11], Lisa Komoroske[12], Camila J. Mazzoni[1]

[1] Department of Evolutionary Genetics, Leibniz-Institut für Zoo- und Wildtierforschung (IZW), Berlin, Germany

[2] Department of Genetics, Evolution, Immunology and Microbiology, State University of Campinas, Campinas, SP, BR

[3] Senckenberg Research Institute, Senckenberganlage 25, 60325 Frankfurt, Germany

[4] Institute of Cell Biology and Neuroscience, Faculty of Biosciences, Goethe University Frankfurt, Max-von-Laue-Str. 9, 60438 Frankfurt, Germany

[5] Universität Potsdam, Brandenburg, Potsdam, Germany

[6] Tree of Life, Wellcome Sanger Institute, Cambridge, CB10 1SA, UK

[7] Faculty of Biosciences and Aquaculture, Nord University, Bodø, Norway

[8] Department of Biological Sciences, Smith College, Northampton MA 01063 USA

[9] Natural Sciences Collegium, Eckerd College, 4200 54 Ave S, St Petersburg, FL, 33711, USA

[10] Department of Ecology and Evolutionary Biology, University of California, Los Angeles, CA 90095

[11] La Kretz Center for California Conservation Science, Institute of the Environment and Sustainability, University of California, Los Angeles, CA 90095

[12] Department of Environmental Conservation, University of Massachusetts Amherst, Amherst, MA 01003

Correspondence to Elisa Ramos: e209572@dac.unicamp.br and Camila J. Mazzoni mazzoni@izw-berlin.de
Address: Laboratório de Genômica Evolutiva. Departamento de Genética, Evolução, Microbiologia e Imunologia, Universidade Estadual de Campinas, Cidade Universitária, 13083970 - Campinas, SP - Brazil

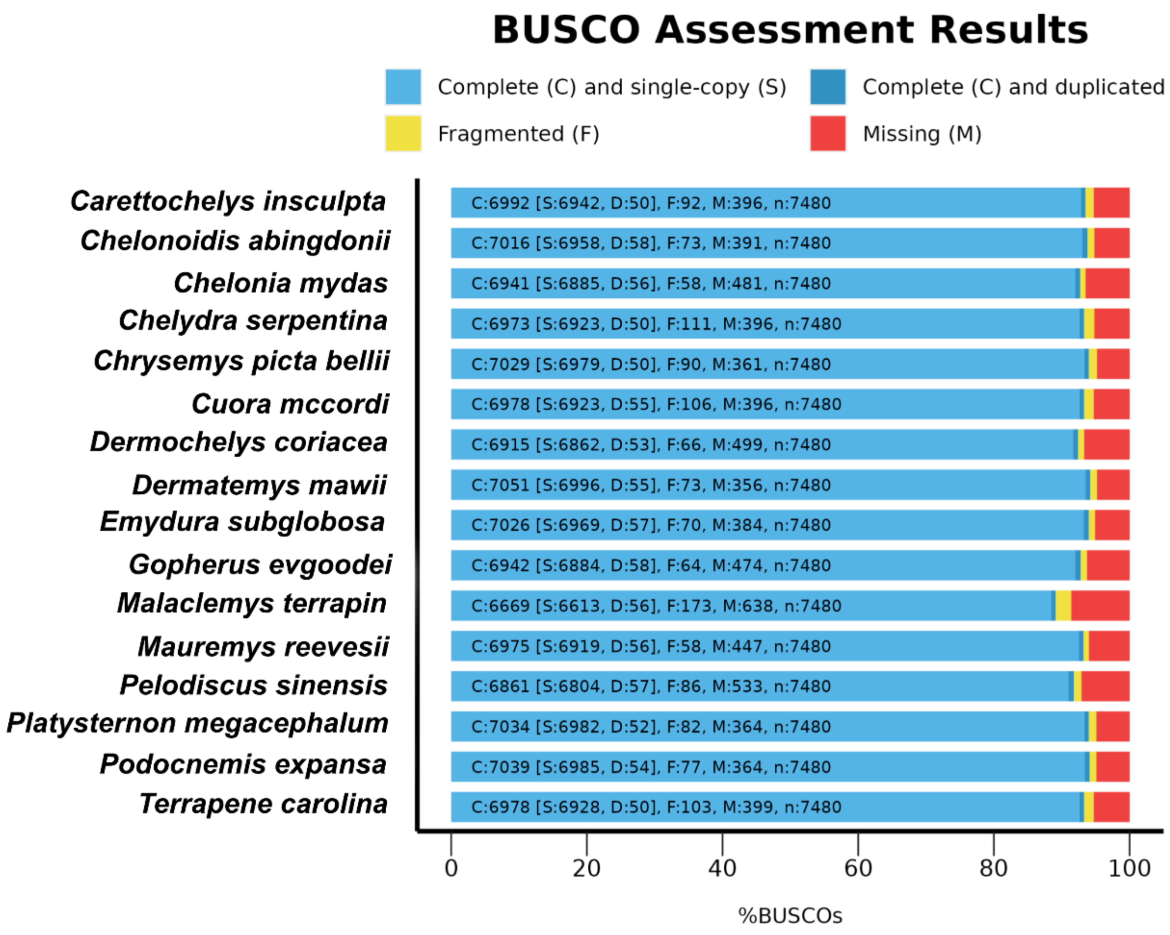

Figure S1. BUSCO scores for the 16 selected turtle genome assemblies retrieved by BUSCOv5 after TOGA analysis using sauropsida_Odb10 database.

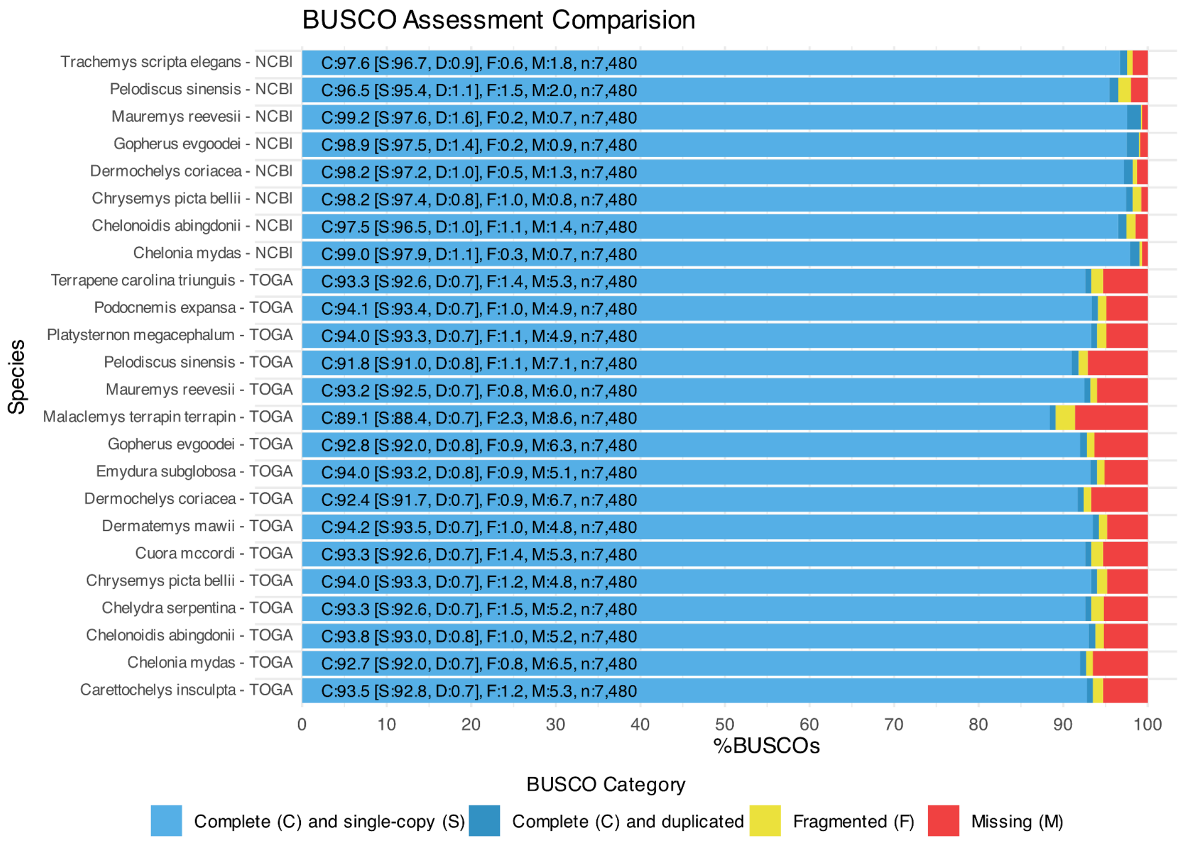

Figure S2. BUSCO scores comparisons between NCBI annotations and TOGA annotation for the 17 selected turtle genome assemblies using sauropsida_Odb10 database.

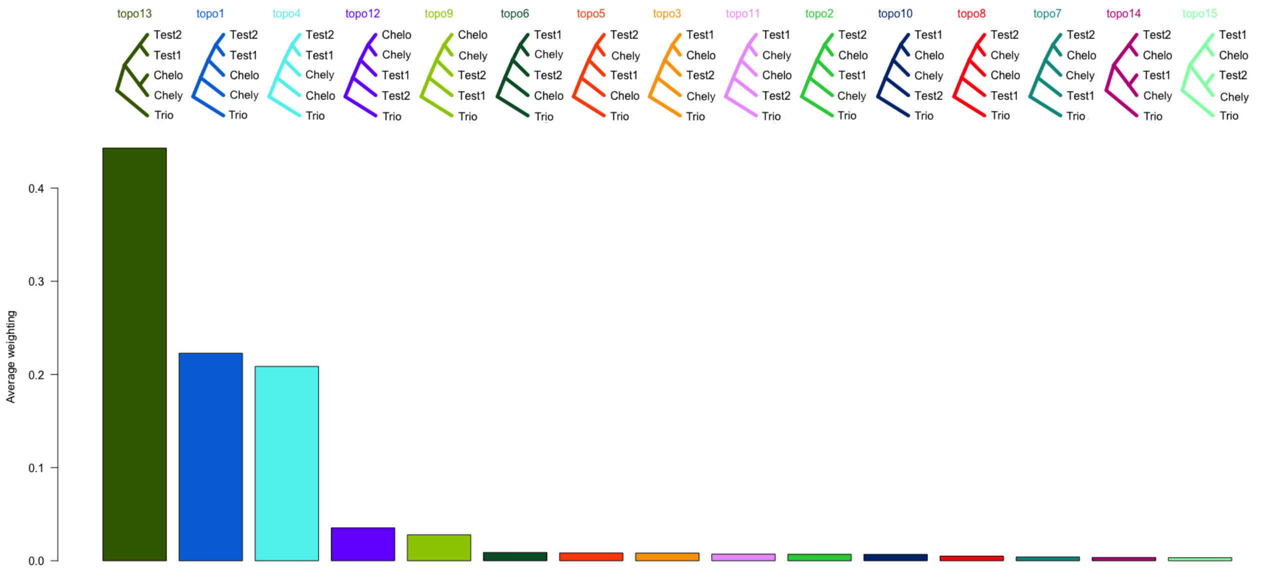

Figure S3. Topology weighting by an iterative sampling of sub-trees (Twisst) for eight turtle species grouped in 5 groups. Topology 3, 10 and 15 groups together the clade of sea turtles and the Testudinoidea1 clade, containing the brackish turtle *Malaclemys terrapin*. Trio=Trionychoidea, Chely=Chelydroidea, Chelo=Chelonioidea, Test1=Testudinoidea1 and Test2=Testudinoidea2.

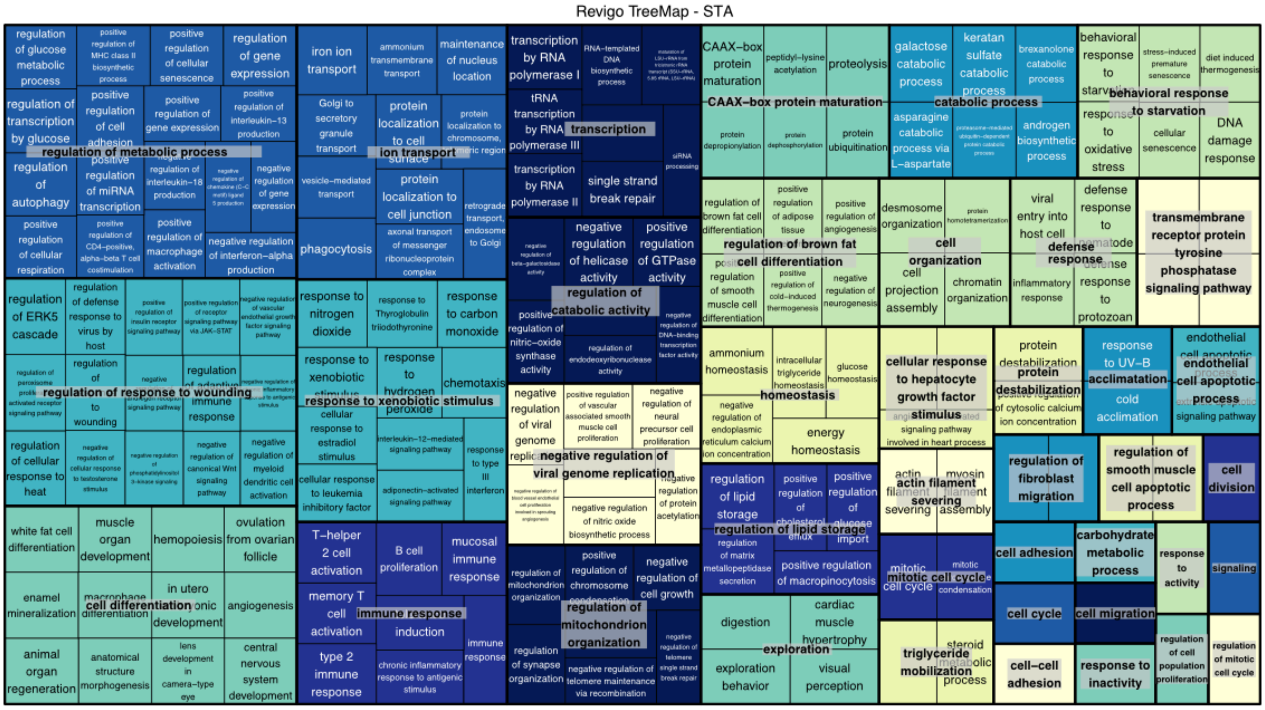

Figure S4. REVIGO Summarised GO terms of genes with evidence of positive episodic selection that are exclusive for stem marine turtle (SMT) estimated with aBSREL.

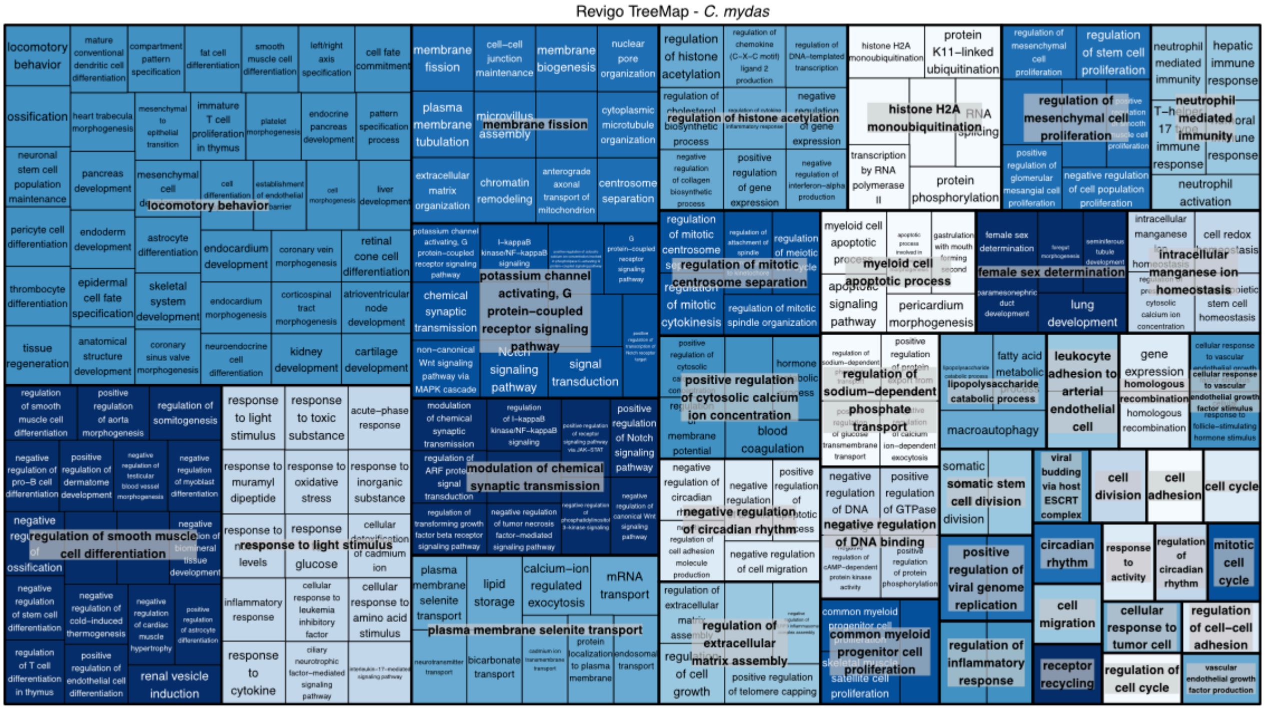

Figure S5. REVIGO Summarised GO terms of genes with evidence of positive episodic selection that are exclusive for *Chelonia mydas* estimated with aBSREL.

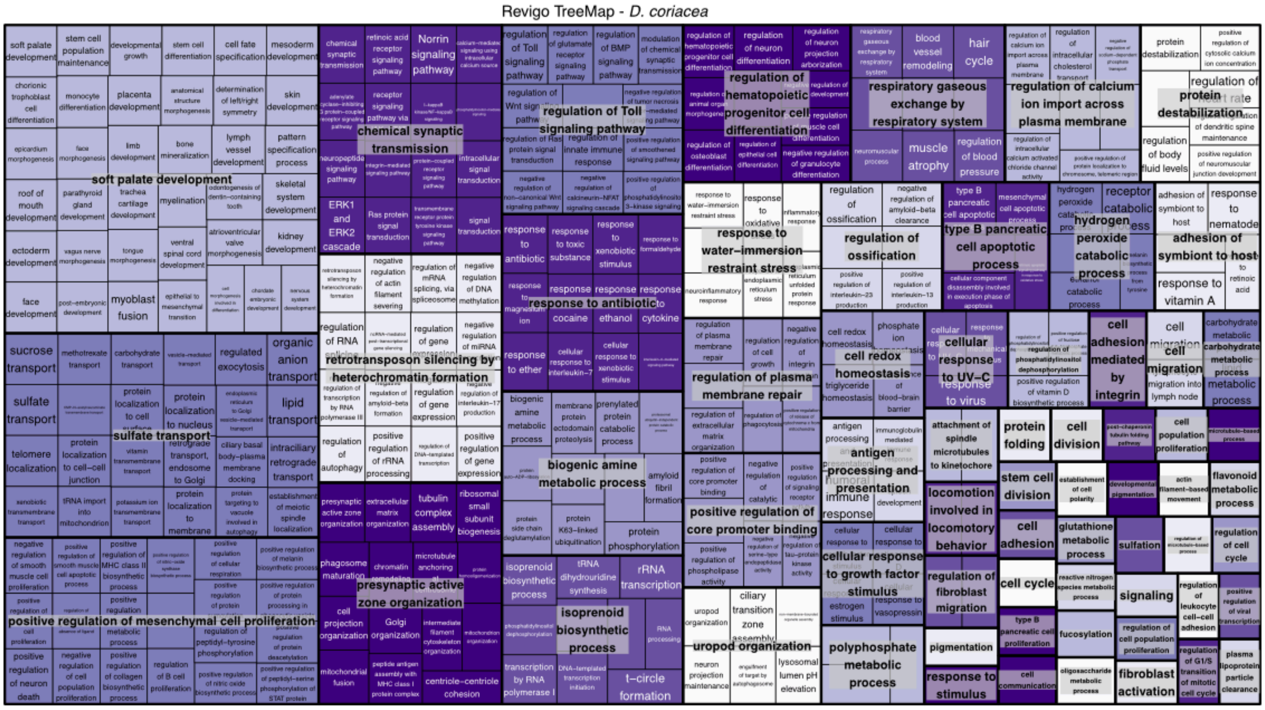

Figure S6. REVIGO Summarised GO terms of genes with evidence of positive episodic selection that are exclusive for *Dermochelys coriacea* estimated with aBSREL.

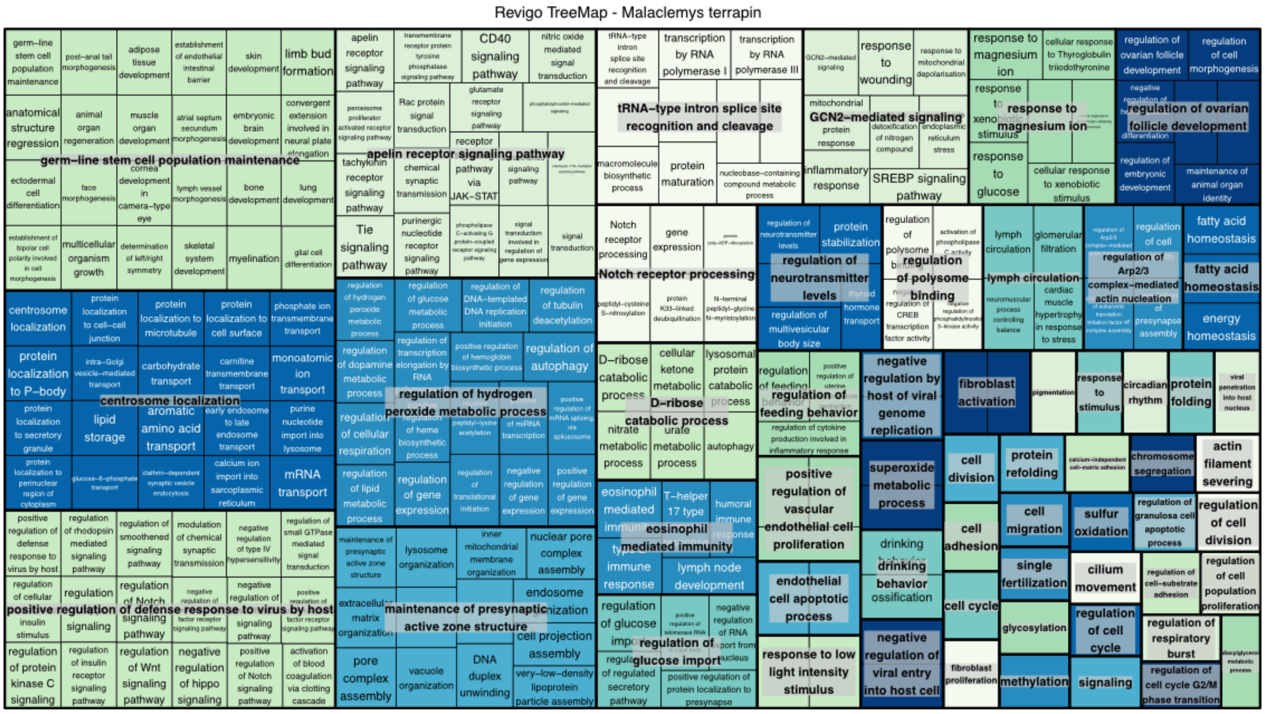

Figure S7. REVIGO Summarised GO terms of genes with evidence of positive episodic selection that are exclusive for *Malaclemys terrapin terrapin* estimated with aBSREL.

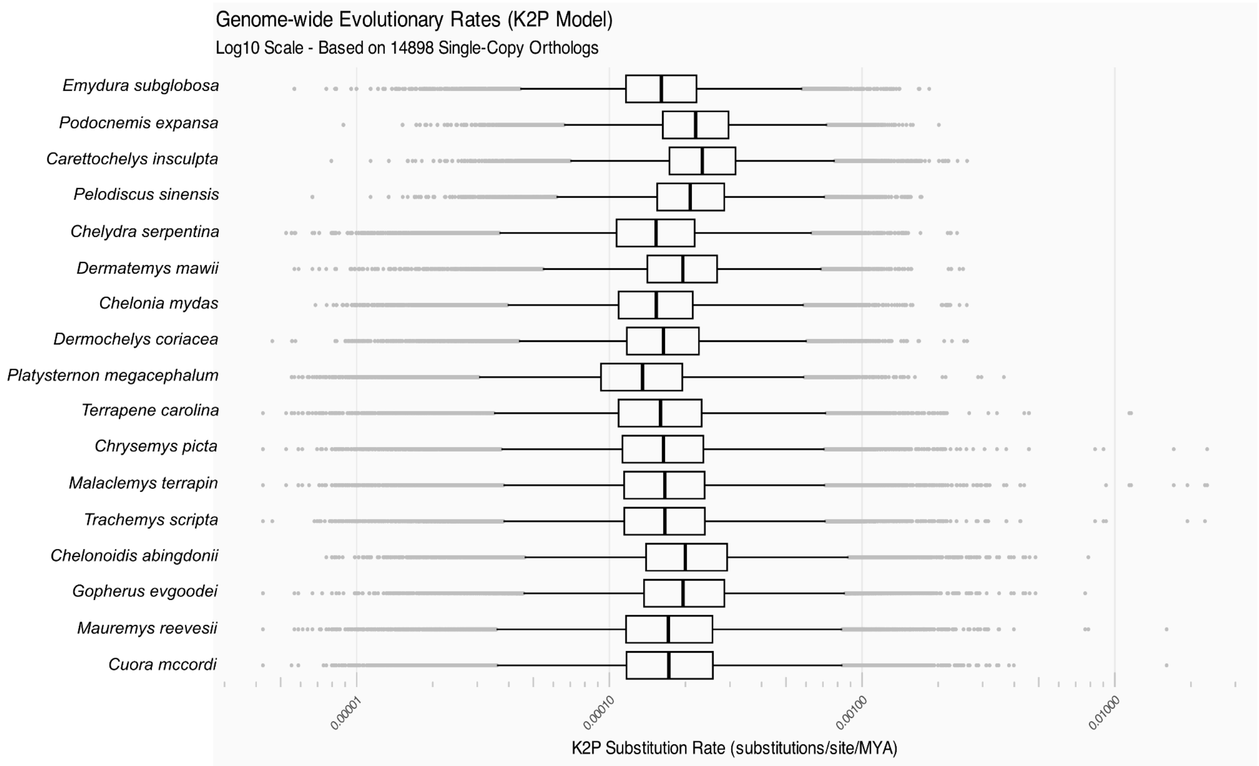

Figure S8. Evolutionary rates in turtles based on 14,898 single-copy orthologous gene alignments. Boxplots show the median with hinges at the 25th and 75th percentile and whiskers extending 1.5 times the interquartile range

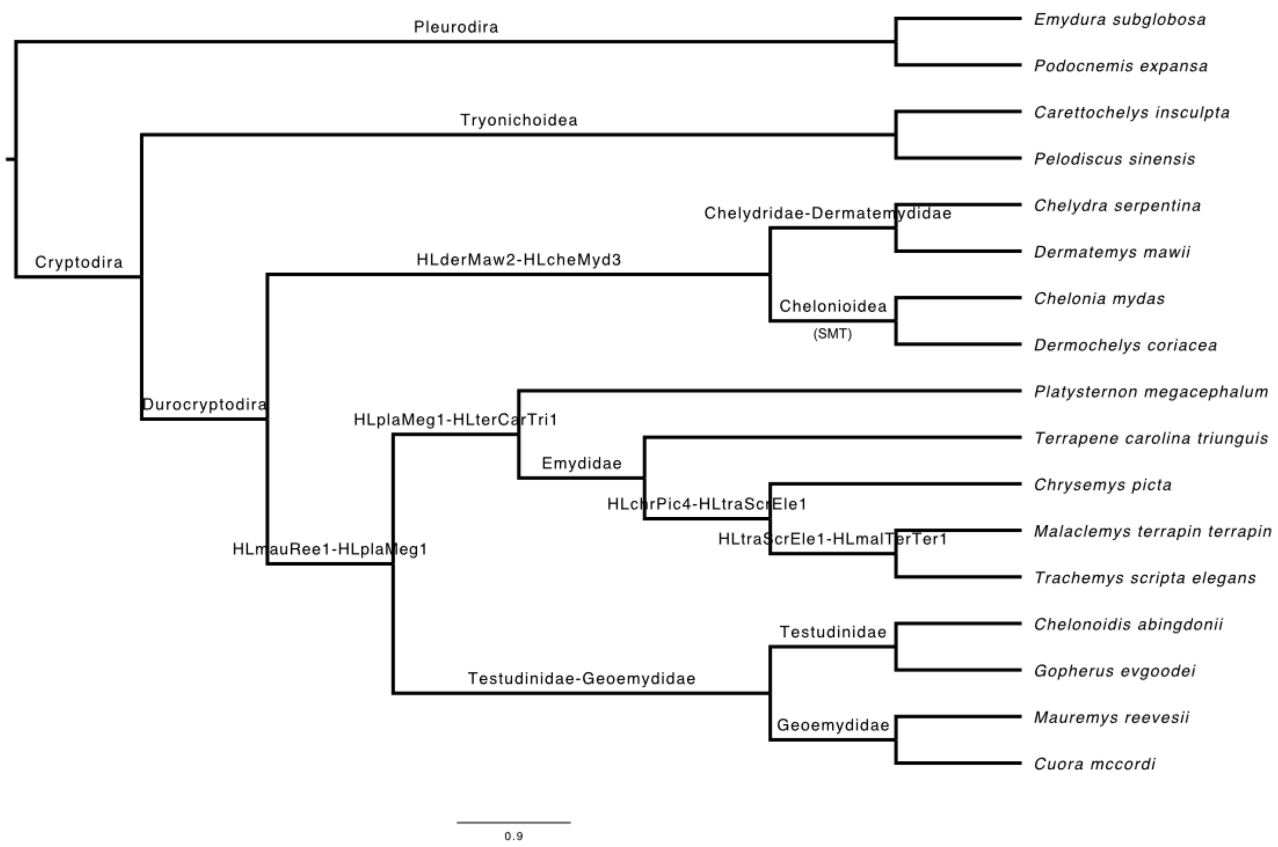

Figure S9. Topology of species tree used in aBSREL analysis showing the names used for each branch.

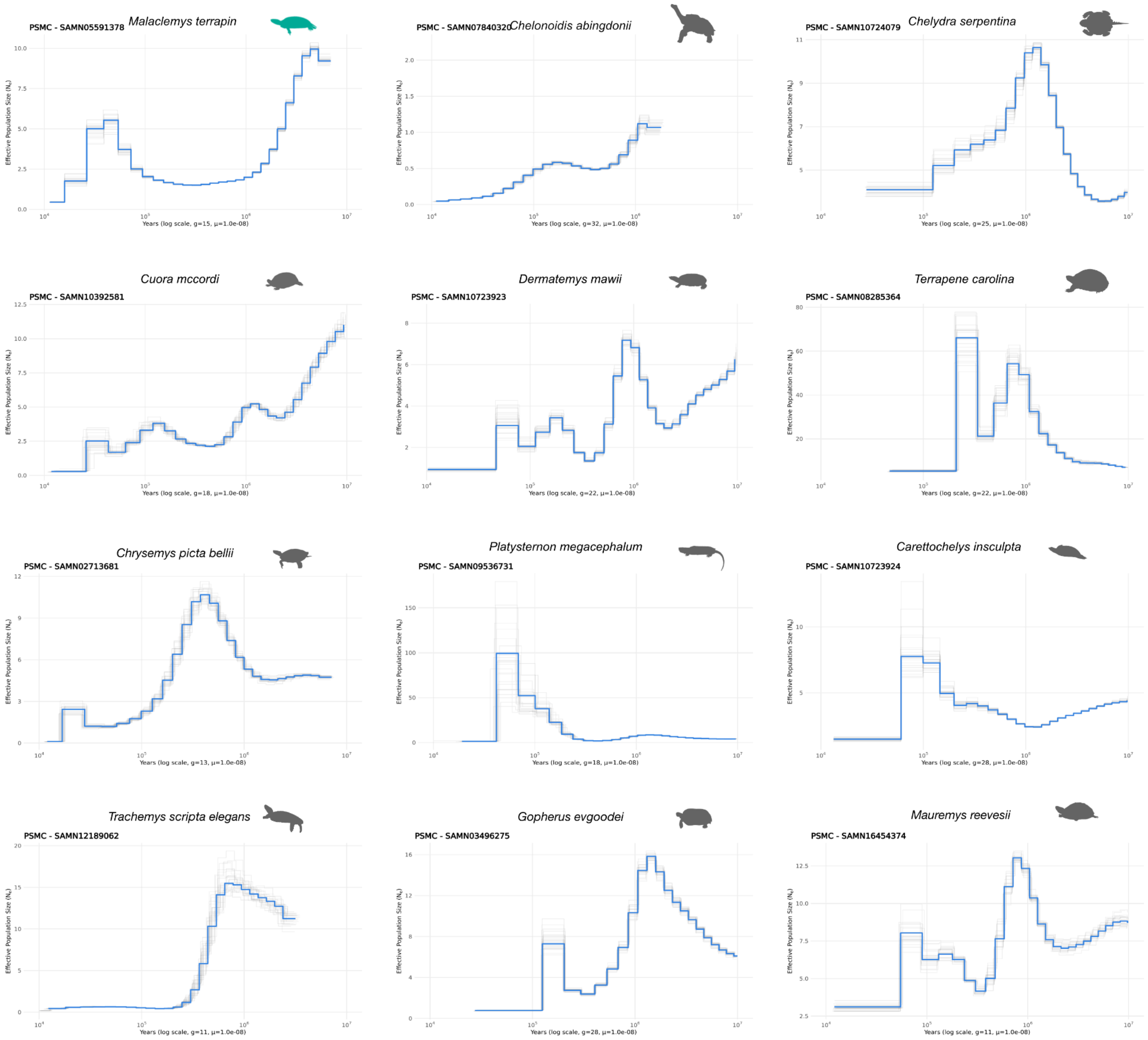

Figure S10. Ancient demographic history for the 12 chelonian reconstructed with default parameters using pairwise sequentially Markovian coalescent (PSMC) plot. Inferred fluctuations in Ne were rescaled assuming different generation times for each species (Supplementary table 24) and $1.0 \times 10{-}8$ per generation mutation rate. Faint lines indicate uncertainty of inferred Ne based on 25 bootstrap replicates.
